# Integrative multiomics analysis reveals host-microbe-metabolite interplays associated with the aging process in Singaporeans

**DOI:** 10.1080/19490976.2022.2070392

**Published:** 2022-05-12

**Authors:** Liwei Chen, Tingting Zheng, Yifan Yang, Prem Prashant Chaudhary, Jean Pui Yi Teh, Bobby K. Cheon, Daniela Moses, Stephan C. Schuster, Joergen Schlundt, Jun Li, Patricia L. Conway

**Affiliations:** a School of Chemical and Biomedical Engineering, College of Engineering, Nanyang Technological University, Singapore; b Nanyang Technological University Food Technology Centre (NAFTEC), College of Engineering, Nanyang Technological University, Singapore; c Department of Infectious Diseases and Public Health, The Jockey Club College of Veterinary Medicine and Life Sciences, City University of Hong Kong, Hong Kong, China; d Office of Education Research, and Physical Education and Sports Science, National Institute of Education, Nanyang Technological University, Singapore; e Epithelial Therapeutics Unit, National Institute of Allergy and Infectious Disease, National Institutes of Health, Bethesda, Maryland, USA; f School of Social Sciences, Nanyang Technological University, Singapore; g Singapore Institute for Clinical Sciences, Agency for Science Technology and Research (A*STAR), Singapore; h Eunice Kenndy Shriver National Institute of Child Health and Human Development, National Institutes of Health, Bethesda, Maryland, USA; i Singapore Centre for Environmental Life Sciences Engineering, Nanyang Technological University, Singapore; j School of Data Science, City University of Hong Kong, Hong Kong, China; k Centre for Marine Science and Innovation, School of Biological, Earth and Environmental Sciences,The University of New South Wales, Sydney, NSW, Australia

**Keywords:** Microbiome, fecal metabolome, multi-omics, integrative analysis, aging

## Abstract

The age-associated alterations in microbiomes vary across populations due to the influence of genetics and lifestyles. To the best of our knowledge, the microbial changes associated with aging have not yet been investigated in Singapore adults. We conducted shotgun metagenomic sequencing of fecal and saliva samples, as well as fecal metabolomics to characterize the gut and oral microbial communities of 62 healthy adult male Singaporeans, including 32 young subjects (age, 23.1 ± 1.4 years) and 30 elderly subjects (age, 69.0 ± 3.5 years). We identified 8 gut and 13 oral species that were differentially abundant in elderly compared to young subjects. By combining the gut and oral microbiomes, 25 age-associated oral-gut species connections were identified. Moreover, oral bacteria *Acidaminococcus intestine* and *Flavonifractor plautii* were less prevalent/abundant in elderly gut samples than in young gut samples, whereas *Collinsella aerofaciens* and *Roseburia hominis* showed the opposite trends. These results indicate the varied gut-oral communications with aging. Subsequently, we expanded the association studies on microbiome, metabolome and host phenotypic parameters. In particular, *Eubacterium eligens* increased in elderly compared to young subjects, and was positively correlated with triglycerides, which implies that the potential role of *E. eligens* in lipid metabolism is altered during the aging process. Our results demonstrated aging-associated changes in the gut and oral microbiomes, as well as the connections between metabolites and host-microbe interactions, thereby deepening the understanding of alterations in the human microbiome during the aging process in a Singapore population.

## Introduction

Aging is a physiological process that is affected by multiple factors from genetics to environmental factors, including healthy diet, physical activity, smoking and alcohol consumption, and age-related chronic health conditions.^1,2^ Considerable attention has been given to the development of effective strategies against aging. Massive evidence indicates that average telomere length (TL) could act as a biomarker for biological aging,^3^ and recent animal studies reveal the connection of the telomere length and the gut microbiota.^4,5^ Moreover, large amount of data have revealed that dietary antioxidant supplementation such as Vitamins C and E, polyphenols, carnosine and lipoic acid, have significant anti-aging effects.^6^ Caloric restriction might prolong lifespan by regulating insulin-like growth factor (IGF) and the mammalian target of rapamycin (mTOR) pathway.^7^ Moreover, gut microbiota-derived metabolites may have important effects on human longevity. For example, metformin is a promising anti-aging molecule and its pro-longevity effect has been found to be indirectly mediated by its influence on bacteria.^8^

It has been well documented that the gut microbiota is closely associated with the aging process.^9^ For example, some studies have indicated that the gut microbial diversity is reduced in elderly subjects,^10,11^ while another study showed that microbial diversity significantly increases with age based on individuals from the American Gut Project.^12^ Furthermore, a cross-sectional study in 367 healthy Japanese subjects from newborns to centenarians revealed that microbial diversity continues to increase until an individual’s twenties, remains stable during adulthood, and then increases again during the elderly stage.^13^ These studies indicated that the patterns of the compositional changes of gut microbiota are highly variable across different studies and populations. Others have revealed significant differences in the abundance of specific bacteria. It has been reported that elderly people have a higher abundance of Bacteroidetes but harbor fewer bifidobacteria, *Clostridium cluster* IV, *Blautia* and *Ruminococcus* compared to young people.^14–17^

In contrast to many studies investigating the relationships between the gut microbiota and age, the associations of oral microbiota with age have only recently started to attract the attention of researchers. Percival *et al*. found that the abundance of *Actinomyces* species, especially *Actinomyces naeslundii* and *Actinomyces oris*, are significantly higher in the supragingival biofilm of subjects over 60 years of age.^18^ Another study demonstrated that compared with young subjects, older people have a higher prevalence of enteric bacilli and *Pseudomonas* species.^19^ Accumulating evidence has revealed that the decline in host immunity and general functionality during aging are partially attributed to the composition shift of the oral microbiota and the feedback loop of the transition of oral bacteria to the gastrointestinal tract.^13,20^ This highlights the necessity to investigate the role of the oral microbiome during the aging process.

Although age-associated signatures in the gut or oral microbiome have been studied, investigations based on the microbiome at a single body site may hinder a systemic understanding of the microbial influence on the aging process. Moreover, interactions between the gut and oral microbiomes have drawn closer attention to host physiology. It has been shown that the oral microbiome could influence host physiology and disease development by modulating the overall composition of the gut microbiome.^21,22^ Moreover, Schmidt et al. identified extensive oral-gut transmission in 470 individuals from five countries and demonstrated increased transmission in patients with bowel cancer and rheumatoid arthritis compared to healthy controls.^23^ Notably, Iwauchi *et al*. found that some oral bacteria are more prevalent in the feces of elderly subjects than in the feces of adult subjects.^24^ This finding demonstrated that the bacterial transition from oral to gut was affected by age. Thus, it is of great value to combine the gut and oral microbiomes to comprehensively investigate the characteristics of human microbial communities with age. Additionally, the gut metabolome is considered the complete set of metabolites that are secreted or modified by the gut microbiome and/or the host, thereby providing valuable information about the metabolic activity of microbes, the host and their co-metabolism.^25^ Therefore, it is imperative to combine metagenomics, metabolomics and key host phenotypic parameters to reveal how host-microbe-metabolite interplays relate to the aging process. This is particularly relevant for Southeast Asian countries, including Singapore, with rapidly increasing populations of elderly.

In this study, for the first time, we characterized the gut and oral microbiomes and the gut metabolome, stratified by two age stages, from 62 healthy male Singaporeans (32 young adults and 30 elderly) by employing shotgun metagenomic sequencing and non-targeted metabolomics approaches. We identified age-related signatures in the microbial compositions and functions and gut metabolites. Furthermore, our integrative analysis based on multiomics datasets revealed potential host-microbe-metabolite interactions, which provide novel insights into the effect of microbiota on aging. This study could help deepen our understanding of the mechanisms underlying age-associated changes in the human microbiome.

## Results

### Overview of gut and oral microbiome compositions in the young and elderly groups

**Figure 1.**
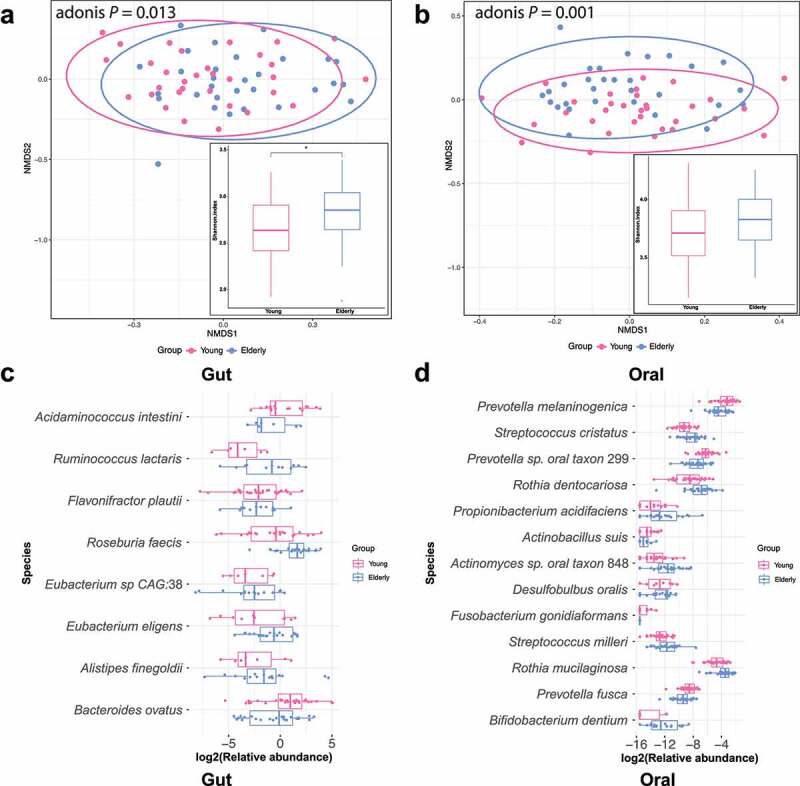
**Age-associated changes in the gut and oral microbiomes**. A & B: Nonmetric multidimensional scaling (NMDS) plots based on the Bray-Curtis dissimilarity matrix illustrate that the beta diversity was significantly different between the elderly and young groups. The boxplot shows the alpha diversity (Shannon index) of the gut (a) or oral (b) microbiota. Higher levels of alpha diversity were observed in the elderly gut and oral microbiota, but the difference was not statistically significant in oral samples (Wilcoxon test: gut microbial community: *p* = .038, oral microbial community: *p* = .146). *p < .05 by Wilcoxon signed-rank test between the elderly and young groups. C & D: Significantly altered species (adjusted *p*-value < 0.1) of the gut (or oral) microbiota community in the elderly group compared to the young group.

We profiled the structures of the gut and oral microbiomes based on shotgun metagenomic data at different taxonomic levels (See Supplementary Figure S1A and S2A, and Supplementary Table S1 for more information). We further compared the overall community compositions of subjects based on Bray-Curtis distance. This analysis revealed that the gut and oral community compositions of elderly subjects were significantly different from those of young people (Figures 1(a, b), gut: Adonis test *p* = .013; oral: Adonis test *p* = .001). Moreover, elderly people had higher alpha diversity (Shannon index) of the gut and oral microbiomes than young people (Figure 1(a, b), gut: Wilcoxon test *p* = .038; oral: Wilcoxon test *p* = .146), which is consistent with a previous report of the American Gut Project that gut microbiota diversity increased with age.^12^ Similarly, a higher species richness was also shown in the elderly gut and oral microbiota, but the difference was not statistically significant in oral samples (Supplementary Figure S1B and S2B, gut: Wilcoxon test *p* = .001; oral: Wilcoxon test *p* = .994).

### Age-related changes in the gut and oral microbiotas

We performed differential abundance analysis by comparing the species-level relative abundance between the elderly and young groups. Eight differentially abundant species (FDR *p* < .1, prevalence > 30%) were identified in the gut microbiome between the two age groups. As shown in Figure 1c, 5 species, namely, *Ruminococcus lactaris, Roseburia faecis, Eubacterium* sp. CAG 38, *Eubacterium eligens*, and *Alistipes finegoldii*, had higher abundances in the elderly subjects than in the young subjects. In contrast, 3 species, namely, *Flavonifractor plautii, Bacteroides ovatus* and *Acidaminococcus intestine*, had lower abundances in elderly subjects. The increased level of *Eubacterium eligens* was associated with elderly subjects in a healthy Thai population study.^14^

In the oral microbiome, we identified 13 differentially abundant species in elderly individuals compared to young individuals (FDR *p* < .1, prevalence > 30%) (Figure 1d), where there were no shared differentially abundant species with the gut microbiome. Of the 13 species, some species including *Streptococcus milleri, Rothia mucilaginosa, Rothia dentocariosa* and *Desulfobulbus oralis*, had greater abundances in elderly people. Conversely, 5 species, including *Prevotella melaninogenica, Prevotella fusca, Prevotella* sp. oral taxon 299, *Fusobacterium gonidiaformans* and *Actinobacillus suis*, showed lower abundance in elderly subjects.

### Associations between the gut and oral microbiotas during aging

The relationship between the gut and oral microbiomes was investigated to obtain a comprehensive picture of the associations of human microbial communities at different body sites during aging. First, the community similarity analysis evaluated using the Bray-Curtis distance revealed a clear separation between gut and oral samples (Anosim test *p* = .001, Supplementary Figure S3), which indicated that the gut and oral communities have distinct microbial communities, in line with a previous study.^26^ Moreover, the Bray-Curtis distances between gut and oral microbial communities in the elderly group were significantly larger than those in the young group (Wilcoxon test: *p* = .020) (Supplementary Figure S4), which suggests that the gut and oral microbial communities became more diversified with aging.

The species presence/abundance of the gut and oral samples between young subjects and elderly subjects was compared. A total of 82 species were detected in both the oral and gut microbial communities. Of these species, *Escherichia coli* and *Streptococcus infantarius* showed significantly differential abundance between gut and oral communities in the young group only, whereas 13 species with significantly differential abundance, including *Lactococcus lactis* and *Enterococcus faecium*, were solely found in the elderly group (Supplementary Figure S5 and Table S2). Moreover, we observed that *Lactococcus lactis* and *Enterococcus faecium*, both regarded as potentially beneficial, were present in some young gut samples but were absent from all elderly gut samples. These results suggested that the oral-gut cross-talk of beneficial bacteria varies during the aging process.

**Figure 2.**
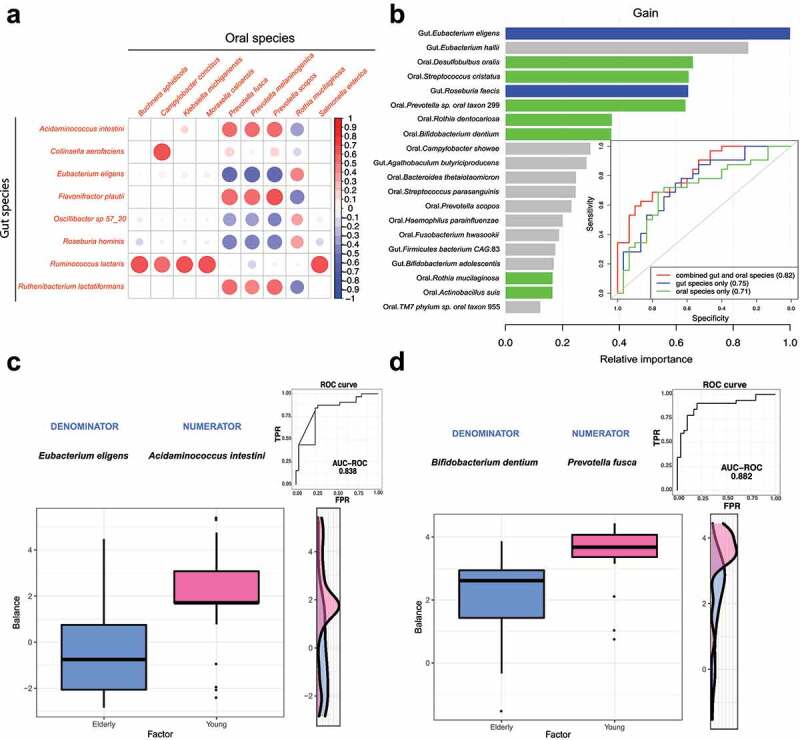
**Gut and oral microbiome signatures differentiate elderly and young groups**. A. Association analysis between gut and oral species using sPLS-DA. Only features showing strong significant correlations (|correlation| > 0.4 and FDR < 0.05) in any of the pairwise associations were used for the visualization. B. Performance of the XGBoost model for the discrimination between the elderly and young groups using species from both gut and oral communities. The bar plot shows the feature importance scores of the top 20 most important features, which were generated by XGBoost. The gut and oral species that had differential abundance between the elderly and young groups are highlighted (gut: blue bar; oral: green bar). C&D: application of the *selbal* algorithm to gut (c) and oral (d) microbial communities to identify the microbial signatures predictive of the young and elderly groups. The box plots show the balance value distribution in the two age groups. The right part of each figure contains the ROC curve with its AUC value and the density curve.

Sparse partial least squares discriminant analysis (sPLS-DA) was carried out to identify highly correlated gut and oral species that allowed differentiating elderly and young subjects (Figure 2a and Supplementary Table S3). We identified 25 pairwise associations (|correlation| > 0.4 and FDR < 0.05) consisting of 8 gut and 9 oral species. Oral *Prevotella* species (*Prevotella fusca, Prevotella melaninogenica* and *Prevotella scopos*) showed 16 (64%) associations with gut species. Notably, we found that several species that had differential abundance between the elderly and young groups were correlated with each other (Figure 2a). *Prevotella melaninogenica* was positively correlated with gut *Acidaminococcus intestini* (correlation = 0.532) and *Flavonifractor plautii* (correlation = 0.577) and negatively correlated with *Eubacterium eligens* (correlation = −0.563). However, oral *Rothia mucilaginosa* showed negative associations with gut *Acidaminococcus intestini* (correlation = −0.399), *Flavonifractor plautii* (correlation = −0.462) and *Eubacterium eligens* (correlation = 0.409). Together, these results imply that the oral-gut cross-talk of key microbial signatures is linked with the aging process.

### Microbiome signatures differentiating between the two age groups

We investigated whether the microbial signatures of the gut and oral microbiomes can be integrated into a machine learning predictive model to differentiate the two age groups. To this end, we built an XGBoost model based on the relative abundance profiles of gut and oral species to classify elderly samples and young samples with 5-fold cross-validation (Figure 2b). The model performance was evaluated using the sensitivity, specificity, accuracy and area under the receiver operating characteristic (ROC) curve (AUC). Using the relative abundance of gut species alone, the model achieved the highest accuracy of 0.71 (sensitivity: 0.88, specificity: 0.53) and an AUC of 0.75. The model based on oral species alone showed similar predictive performance (the highest accuracy value = 0.73 when sensitivity = 0.72 and specificity = 0.73, AUC = 0.71). Combining gut and oral species only slightly boosted the best performance (the highest accuracy value = 0.74 when sensitivity = 0.8 and specificity = 0.69, AUC = 0.82), which indicated strong associations and mutual information between gut and oral microbiome signatures.

We further identified the top 20 most informative features contributing to this model, including 2 gut species (*Eubacterium eligens, Roseburia faecis*) and 7 oral species (*Actinobacillus suis, Bifidobacterium dentium, Desulfobulbus oralis, Prevotella* sp. oral taxon 299, *Streptococcus cristatus, Rothia mucilaginosa* and *Rothia dentocariosa*), whose abundances were different between the elderly and young groups. This indicates that such microbial signatures presented highly predictive capability in distinguishing the two age groups (Figure 2b and Supplementary Table S4).

Subsequently, a *selbal*^27^ analysis was performed to identify the best balance of species, indicated by the geometric means of the relative abundances of several taxa associated with age groups. In the gut microbiome, the *Acidaminococcus intestine/Eubacterium eligens* balance was capable of differentiating the two age groups (cross-validation adjusted AUC = 0.838) (Figure 2c). Similarly, the balance consisting of *Prevotella fusca* (numerator) and *Bifidobacterium dentium* (denominator) showed strong predictive ability in the oral microbiome (cross-validation adjusted AUC = 0.882) (Figure 2d). These results indicated that bacterial interactions are strongly associated with the aging process.

### Age-related changes in the metabolome of the gut microbiota

**Figure 3.**
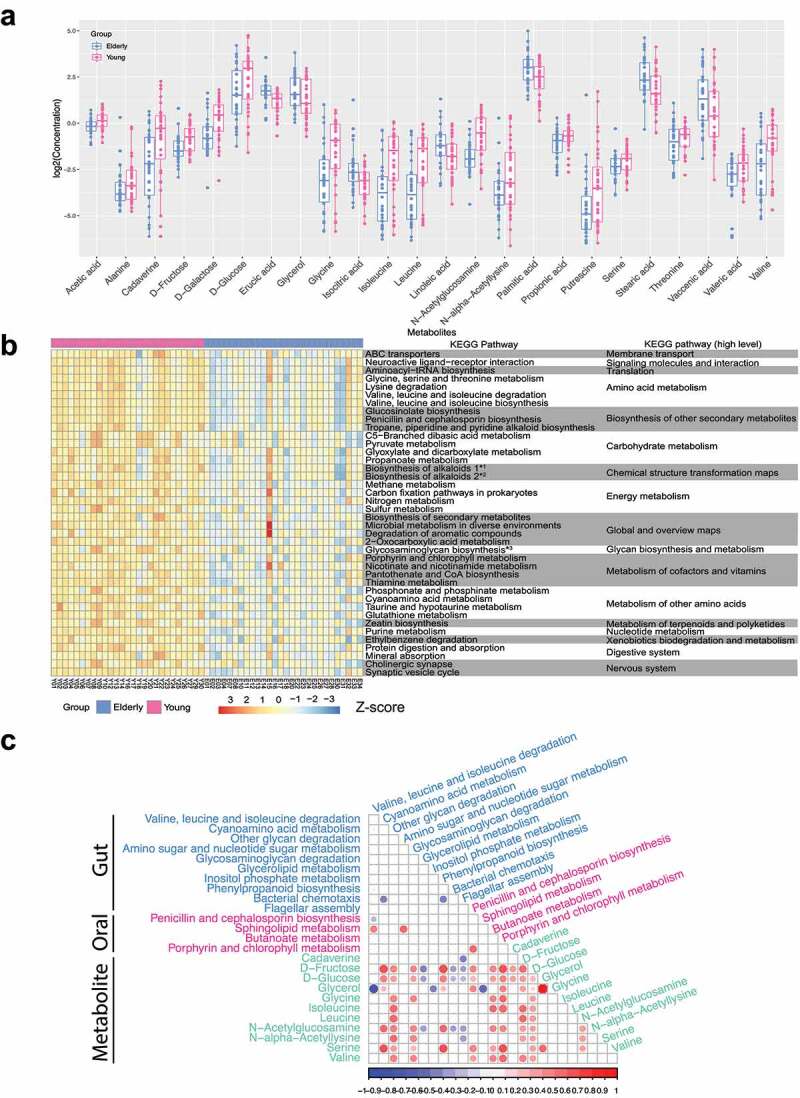
**Age-related changes in the gut metabolome**. A. Metabolites showing differential abundance between the elderly and young groups. B. Heatmap of metabolic pathways showing differential activity (measured as PAPi score) between the elderly subjects and young subjects. For visualization, the PAPi scores were log-transformed and then centered and scaled to a mean of 0 and a standard deviation of 3. The FDR-adjusted *p*-value threshold was set as 0.01. *1: Biosynthesis of alkaloids derived from ornithine, lysine and nicotinic acid; *2: Biosynthesis of alkaloids derived from the shikimate pathway. C. Integrative analysis of gut and oral metagenomic pathways and gut metabolites using sPLS-DA. Only features showing correlations of > 0.4 or < −0.4 in any of the pairwise associations were used for the visualization. The significance level was set at a *p*-value < 0.05.

To investigate the associations between the fecal metabolome and the aging process, gas chromatography-mass spectrometry (GC-MS)-based non-targeted metabolomics profiling of fecal samples was carried out. Of the 117 detected features, 44 metabolites (Supplementary Table S5) were identified using the NIST library with a similarity index above 90%. A total of 24 metabolites showed significant differences (FDR p value<.05) between the elderly and young groups (Figure 3a and Supplementary Table S6). Overall, long-chain fatty acids (LCFAs), including palmitic acid, stearic acid, linoleic acid, erucic acid and vaccenic acid, were present at significantly higher levels in elderly subjects. Conversely, short-chain fatty acids (SCFAs), such as acetic acid and propionic acid, were lower in elderly people, consistent with previous reports that SCFAs gradually declined during aging.^28,29^ Branched-chain amino acids (BCAAs: valine, leucine, isoleucine) were found to be significantly decreased in elderly subjects, which was in accordance with studies showing that BCAAs had a beneficial effect on aging.^30,31^ In addition, other amino acids, such as serine and threonine, were also present at lower levels in elderly subjects. These results highlight that fatty acids and amino acids are the two important metabolic components associated with the aging process.

The pathway activity profiling (PAPi)^32^ algorithm was utilized to quantify the potential metabolic pathway activities based on metabolomic profiles (Supplementary Table S7). In total, 69 metabolic pathways presented significantly different activities between the elderly and young groups, with an FDR-adjusted *P*-value < 0.05 (Figure 3b and Supplementary Figure S6). The majority of the differential metabolic pathways in the fecal metabolome were depleted in elderly subjects. Specifically, the pathways associated with amino acid and carbohydrate metabolism were less active in the elderly subjects than in the young subjects (Figure 3b). The decrease in carbohydrate metabolic activities in the elderly is known as one hallmark of the aging process.^33^ Moreover, other under-represented pathways in the elderly subjects were related to cofactors and vitamin and nucleotide metabolism (Supplementary Figure S6). Several Kyoto Encyclopedia of Genes and Genomes (KEGG) pathways associated with lipid metabolism had higher activity in elderly people, including “Biosynthesis of unsaturated fatty acids (ko01040)”, “Fatty acid elongation (ko00062)”, “Glycerolipid metabolism (ko00561)”, “Fatty acid biosynthesis (ko00061)”, and “Linoleic acid metabolism (ko00591)” (see Supplementary Figure S6). Disorders of lipid metabolism are another feature of aging.^34^ Together, we identified vast differences in fecal metabolites and metabolic pathways between elderly and young individuals, with some key age-associated metabolomic features, including carbohydrate and lipid metabolism.

### Age-associated functional capability of the gut microbiome, the oral microbiome and the gut metabolome

As fecal metabolomics provides a complementary functional readout of microbial metabolism,^35^ we explored the potential links of the age-associated alterations in functional profiling among the gut microbiome, the oral microbiome and the gut metabolome from different perspectives. First, we identified activities of KEGG pathways that differed between elderly and young samples within each -omic dataset (gut microbiome, oral microbiome or gut metabolome) (Supplementary Figure S8). The pathway enrichment analysis revealed that the enrichment/depletion of age-associated gut microbiome-derived pathways was mostly inconsistent with those in the oral microbiome. The pathway “Porphyrin and chlorophyll metabolism (ko00860)” involved in cofactor and vitamin metabolism was the only pathway that was significantly altered in both the gut and oral microbiomes (Supplementary Figure S8, gut: Wilcoxon test *p* = .003; oral: Wilcoxon test *p* = .020). This pathway also significantly changed in the gut metabolomics data (Wilcoxon test *p* = 1.630e-5), which suggested that cofactors and vitamin metabolism might be strongly associated with the aging process.

In addition, we evaluated the concordance between the gut microbiome and metabolome by examining the common enriched functional pathways. Specifically, we identified 5 differentially enriched pathways shared by the gut microbiome and metabolome. Four pathways significantly under-represented in the elderly gut microbiome and metabolome compared to young subjects were “Valine, leucine and isoleucine biosynthesis (ko00290)”, “Starch and sucrose metabolism (ko00500)”, “Amino sugar and nucleotide sugar metabolism (ko00520)” and “Cyanoamino acid metabolism (ko00460)”. In contrast, “Glycerolipid metabolism (ko00561)” was more abundant and active in the gut microbiome and metabolome of elderly people. Together, the results of functional profiling demonstrated that human microbiomes at different body sites and the gut metabolome all contribute to age-associated functions, thereby underlining the importance of the profiling of multiomics data.

Subsequently, sPLS-DA analysis was applied to explore the relationships among gut metabolites, oral KEGG pathways and gut KEGG pathways. There were 52 pairwise associations consisting of 11 gut metabolites, 4 oral microbial functional pathways and 10 gut microbial functional pathways with a cutoff of *p*-value ≤ 0.05 and |correlation| ≥ 0.4 (Figure 3c and Supplementary Table S9). In the gut microbial pathways, “Other glycan degradation (ko00511)” showed the largest number of associations with other pathways/metabolites. “Porphyrin and chlorophyll metabolism (ko00860)” had the most frequent associations in the oral microbial pathways. D-Fructose was the most connected metabolite (16 links in total). The links of gut metabolites with the microbial functional pathways comprised 42 (80.77%) pairwise associations. D-Fructose and D-Glucose were both positively correlated with “Cyanoamino acid metabolism (ko00460)”, “Other glycan degradation (ko00511)”, “Glycosaminoglycan degradation (ko00531)”, “Phenylpropanoid biosynthesis (ko00940)”, and “Butanoate metabolism (ko00650)” and negatively correlated with “Glycerolipid metabolism (ko00561)”, “Bacterial chemotaxis (ko02030)”, and “Flagellar assembly (ko02040)”. Our results revealed strong connections between gut metabolites and the metabolic functional capabilities of the microbiota.

### Integrative analysis of the gut and oral microbiomes, metabolomes and phenotypic parameters

**Figure 4.**
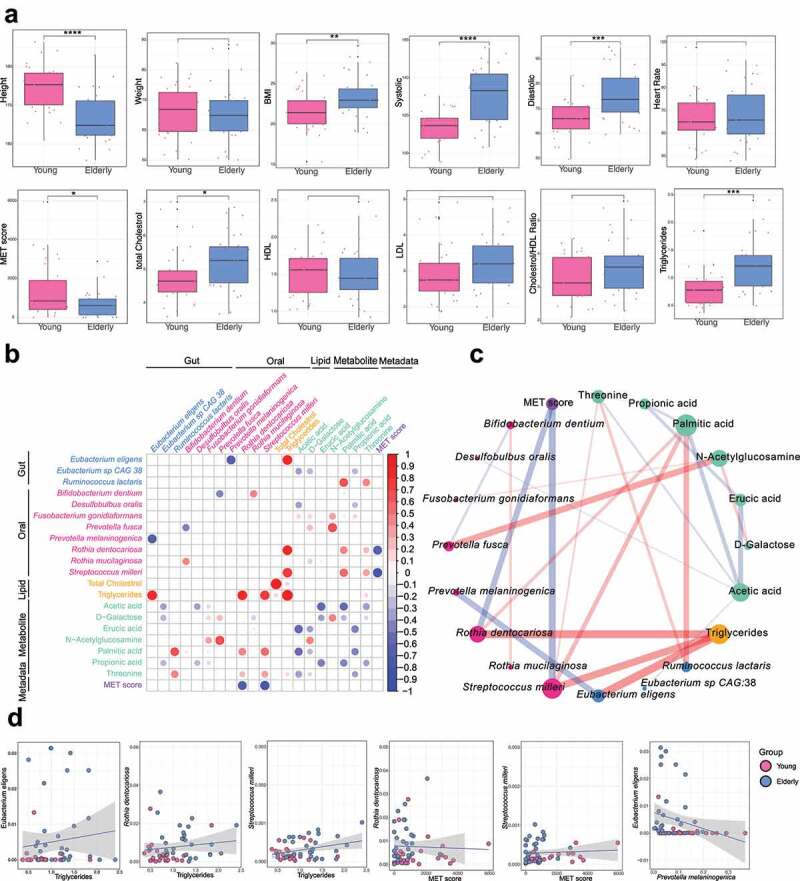
**Integrative analysis of gut and oral microbiome, metabolome and phenotypic features using sPLS-DA**. A. Comparison of phenotypic characteristics between the elderly and young groups. Wilcoxon test: * *p* < .05; ** *p* < .01; *** *p* < .001. B. Integrative analysis of gut and oral microbiome, metabolomics and metadata features using sPLS-DA. Differential features from the multiomics datasets were used for sPLS-DA analysis. Only features showing correlations of > 0.3 or < −0.3 in any of the pairwise associations were used for the visualization. The significance level was set at a *p*-value < 0.05. C. Network visualization of the integration between multiomics datasets. The edges represent significant correlations of > 0.3 or < −0.3 between features. Red edges indicate positive correlations, and blue edges indicate negative correlations. The edge thickness and transparency are proportional to the absolute value of the correlation coefficient. The node size scales with the sum of absolute values of the correlation coefficient. Different node colors indicate different omics datasets (gut: blue; oral: red; metabolite: lime green; metadata: purple; lipid: Orange). D. Scatter plot showing the associations between the abundances of multiomics features.

The anthropometric and clinical parameters, including height, weight, body mass index (BMI), blood pressure level and heart rate (Supplementary Table S10) in the 62 individuals were extensively characterized. Significant increases in BMI and systolic and diastolic blood pressure levels were observed in the elderly group (BMI: Wilcoxon test *p* = .0065; systolic: Wilcoxon test: *p* = 2.9e-5; diastolic: Wilcoxon test: *p* = .00034,) Figure 4a. We also examined the intensity of physical activity measured as the metabolic equivalents (MET) score for all the participants. As expected, the elderly group had a significantly lower physical activity score than the young group (Wilcoxon test *p* = .043). Moreover, we determined the plasma lipid profiles, including triglycerides, total cholesterol, high-density lipoprotein (HDL) cholesterol, low-density lipoprotein (LDL) cholesterol and cholesterol/HDL ratio (Figure 4a). Significant elevations in total cholesterol and triglycerides were observed in the elderly group (total cholesterol: Wilcoxon test *p* = .021; triglycerides: Wilcoxon test: *p* = .00013). All the aforementioned phenotypic parameters were combined into an independent metadata dataset for the subsequent multiomics data integrative analysis.

We extended the application of sPLS-DA^36^ to multiomics analyses among the gut and oral microbiome, gut metabolome, and phenotypic parameters to identify a highly correlated multiomics and aging-related signature panel. Only features with significant differences between elderly subjects and young subjects were used in this analysis. We identified 25 pairwise associations consisting of 21 features (*p*-value ≤ 0.05 and |correlation| ≥ 0.3, Figures 4(b,c). Triglycerides were only positively correlated with other features, with the highest correlations with gut *Eubacterium eligens* (correlation = 0.819), oral *Rothia dentocariosa* (correlation = 0.812) and oral *Streptococcus milleri* (correlation = 0.758) (Figure 4b,4d and Supplementary Table S11), which suggested that some oral and gut microbes may exert positive effects on triglyceride production. On the other hand, the MET score was only negatively correlated with all the other features, with the strongest negative associations against oral *Streptococcus milleri* (correlation = −0.728) and oral *Rothia dentocariosa* (correlation = −0.664) (Figure 4b,4d and Supplementary Table S11), which implied that these bacteria may be greatly influenced by lifestyle, especially the intensity of daily physical activities. Moreover, oral *Prevotella melaninogenica* was only negatively correlated with the gut species *Eubacterium eligens* (correlation = −0.666) (Figure 4b,4d and Supplementary Table S11). In summary, these potential host-microbe-metabolite associations may provide mechanistic insight into how the microbiome influences host aging.

Within the associations among different omics parameters, the metabolite acetic acid showed the largest number of strong negative correlations (5 connections), while another metabolite, N-acetylglucosamine (GlcNAc), had 3 positive correlations. Acetic acid is one of the predominant SCFAs produced by gut microbiota,^37^ and the amino sugar GlcNAc has an impact on cell surface signal transduction as a key component of bacterial cell wall peptidoglycan.^38^ Therefore, our findings confirmed the mediating effect of the gut metabolome on microbiome-host communication. In addition, the fatty acids acetic acid, propionic acid, erucic acid, and palmitic acid were among the 7 metabolite-derived signatures (Figure 4b), further demonstrating that fatty acids are key metabolites linked to the cross-talk between microbiota and host immunity.

Some previous studies showed that LCFAs could promote inflammation, whereas SCFAs suppress inflammation;^39,40^ thus, we examined the possible links of fatty acids exhibiting significant differences (between elderly and young groups) and reported important proinflammatory mediators,^41,42^ including wide-range C-reactive protein (wr-CRP), interleukin-7 (IL-7), interleukin-8 (IL-8), monocyte chemotactic and activating factor (MCP-1/MCAF), macrophage inflammatory protein-1beta (MIP-1b) and tumor necrosis factor alpha (TNF-a) (data are shown in Supplementary Table S10). Interestingly, erucic acid (a monounsaturated LCFA) showed modest but significant positive correlations with IL-7 (Spearman rho = 0.382, *p* = .007) and IL-8 (Spearman rho = 0.343, *p* = .011) (Supplementary Figure S8 and S9). Valeric acid (SCFA) was negatively correlated with IL-7 (Spearman rho = −0.298, *p* = .04) (Supplementary Figure S10). These results suggested that the fatty acids of the gut metabolome possibly contributed to inflammation through the modulation of IL-7, while further investigation is needed to clarify the mechanism linked with the immune response.

## Discussion

In summary, for the first time, we have characterized the gut and oral microbiomes and fecal metabolome stratified by two age stages in a healthy male Singaporean cohort and further identified age-associated signatures and their potential interactions by integrative analysis of multiomics datasets. More importantly, our study may serve as a valuable resource of the microbiome in Singaporeans, contributing to a deeper understanding of the physiological state of the “normal” microbiome during aging.

Aging is a major public health issue in Singapore since 16.8% of the current population aged 65 years and above, and it is estimated that 23.7% of the population will be above 65 years of age by 2030 [Department of Statistics in Singapore, 2020, Available online: https://www.strategygroup.gov.sg/files/media-center/publications/population-in-brief-2020.pdf/]. Singaporeans may harbor a unique microbiome due to their multicultural society and hybrid dietary habits. Consequently, studying the microbiome profiles and their associations with hosts in the Singapore population could provide further clues about the correlations between aging and different community types.

To address this issue, we offer a framework for the integrated analysis of multiomics data, including microbiome, metabolome and various phenotypic parameters. In this study, the crosslinks among different omics datasets were identified based on sPLS-DA. We assessed the robustness of our sPLS-DA results by calculating Spearman correlation of correlation pairs extracted from the gut-oral association analysis and multi-omics integrative analysis. As a result, a highly positively correlated result was observed (Spearman Rho = 0.964, *p* = 1.307e-70, Supplementary Figure S11), which indicated that our sPLS-DA results are robust to noise from multiple omics data. Moreover, we examined the concordance of the associated pairs identified from the multiomics integrative analysis between Kendall rank correlation coefficient (τ) and Spls-DA correlation. The results indicated that the association strength of Spls-DA and Kendall tau are highly correlated (Spearman Rho = 0.663, p = 4.704e-08, Supplementary Figure S11). Besides, none of the pairs showed an inconsistent direction of association (±) between Kendall rank correlation and Spls-DA correlation. Overall, the evaluation of the consistency between Spls-DA and Kendall’s tau supported that associations identified by Spls-DA analysis were overall robust, verified by the Kendall rank correlation test.

We identified different types of age-related signatures from multiomics datasets. Recently, the impact of cyanobacteria on human health has drawn attention. Cyanobacteria represent a diverse group of organisms that produce potent natural toxins, and many of these bacteria present photosynthetic abilities. A deep-branching clade of cyanobacteria was detected in human gut and linked to human health.^43,44^ It has been shown that the hepatotoxic cyanobacteria may also be associated with host tumorigenesis.^45^ In addition, cyanobacteria extract was found to stimulate an antitumor host response against an oral cancer cell line.^46^ Li et al.^47^ investigated the impact of cyanobacteria from the skin microbial community related to aging. For the skin samples collected from abdomen, lower abundances of cyanobacteria (0.41%) were detected in the young group (19–23 years) compared to that (0.87%) in the elderly individuals (65–74 years). In our dataset, we examined the presence of cyanobacteria in the gut and oral microbial communities. In the gut community, cyanobacteria were absent from the taxonomic profile identified by MetaPhlAn3. Then, we attempted to identify taxonomy profiling using Kraken2, which improved the sensitivity with the cost of a higher false positive rate for low-abundance species.^48^ Based on the taxonomic profiles identified by Kraken2, we found that cyanobacteria were present in >96% of the samples but with extremely low average relative abundance (gut: 0.008% and 0.017% for young and elderly subjects, respectively; oral: 0.009% and 0.007% for young and elderly subjects, respectively). The inconsistent discovery of cyanobacteria between Metaphlan3 and Kraken2 and the extremely low abundance quantified by Kraken2 suggest a potential false discovery of cyanobacteria due to the random and systematic errors introduced during the k-mer mapping in our NGS data. Therefore, more solid evidence, including cyanobacteria verified by different tools, is required to elucidate the potential mechanism of how cyanobacteria interact with the aging process in Singaporeans in future studies.

At species level, the effect sizes, measured as fold change of mean abundance in young vs. elderly, were also considered. As Figure 1c indicates, in the gut community, among the more abundant species in the elderly subjects, *Ruminococcus lactaris* and *Alistipes finegoldii* showed a large effect (|log2-fold-change| > 2). Other species with a lower effect (|log2-fold-change| < 2), such as *Eubacterium* sp CAG:38, *Eubacterium eligens*, and *Roseburia faecis*, were reported to produce short-chain fatty acids, playing important roles in maintaining gut homeostasis and immunity.^49,50^ Conversely, *Acidaminococcus intestini* was observed to have significantly lower abundance in the elderly group with a large effect size (log2-fold-change = −2.60). In the oral community, among 8 species that have higher abundance in elderly subjects, *Bifidobacterium dentium* and *Propionibacterium acidifaciens* were found to have a large effect size (|log2-fold-change| > 2). Other species with lower effects (|log2-fold-change| < 2), including *Streptococcus milleri, Rothia mucilaginosa, Rothia dentocariosa* and *Desulfobulbus oralis*, are opportunistic pathogens that may affect immunocompromised hosts and cause systemic infections.^51–54^ In contrast, 5 species were found to have lower levels in the elderly group. Among these species, *Fusobacterium gonidiaformans* showed the highest effect of difference (log2-fold-change = −2.14), which may be highly relevant to aging. Other species include three *Prevotella* species, namely, *Prevotella melaninogenica, Prevotella fusca* and *Prevotella sp. oral taxon* 299, were implicated to link to periodontitis.^55^

In summary, most of the differentially abundant species between the young and elderly groups were found to be closely associated with immunity and inflammation. The highly different species between the two age groups, defined by the high |log2(fold-change)|, implied more biological relevance. The possible links between inflammation and aging have been explored. For example, several studies have shown that older people tend to have a proinflammatory state, which amplifies systemic and localized changes that contribute to the development of aging-related phenotypes and increases the risk of multiple chronic diseases.^56–58^ Moreover, the species with a large effect (|log2-fold change| > 2) of difference might have high discriminatory power and biological relevance. Therefore, the age-related species that we identified might be helpful to gain mechanistic insight into the role of the species in the aging process and the possible mediating effect of inflammation during this process.

We verified the results of differential abundance analysis using compositional data analysis with ANCOM-BC^59^ and ALDEx2,^60^ which explicitly account for the compositional nature of microbiome data. By using ANCOM-BC, in the gut community, a total of four species were identified with FDR *p* < .25. Two species (*Eubacterium eligens*, FDR *p* = .002; *Flavonifractor plautii*, FDR *p* = .152) were also included in the list of differentially abundant species we identified. In the oral community, 13 species were identified with FDR *p* < .25, of which two species (*Bifidobacterium dentium*: FDR *p* = .045; *Actinobacillus suis*: FDR *p* = .050) were differentially abundant by using IndVal test. Similarly, as ALDEx2 was selected for differential abundance analysis, in the gut community, 8 species were identified with FDR p < .25. Five species (*Bacteroides ovatus*, FDR *p* = .131; *Eubacterium eligens*, FDR *p* = .053; *Roseburia faecis*, FDR *p* = .071; *Flavonifractor plautii*, FDR *p* = .084; *Acidaminococcus intestini*, FDR *p* = .139) were included in the list of DA species identified by the IndVal test. In contrast, only *Rothia dentocariosa* (FDR *p* = .195) showed differential abundance with FDR *p* < .25 in the oral community. This species was also present in our results. Taken together, these results demonstrate the robustness of our results of the differential abundance analysis and the key species, such as *Eubacterium eligens* and *Flavonifractor plautii*, can be verified with software based on compositional data analysis. Additionally, the balance consisting of gut species *Eubacterium eligens* and *Acidaminococcus intestini* showed a strong predictive ability to differentiate young and elderly groups in the *selbal* analysis,^27^ another compositional data analysis, confirming the potentially important roles of these two key species during aging.

*Eubacterium eligens* stands out as an important age-related gut microbial signature for discriminating between the two age groups. The species exhibited significantly different abundance between elderly and young groups. In addition, *Eubacterium eligens* showed a high predictive power in the XGBoost model, allowing differentiation of the two age groups (Figure 1e). As a butyrate-producing bacterium, *Eubacterium eligens* plays important roles in maintaining gut homeostasis, immunity maintenance and anti-inflammation.^49,50^ However, the abundance of *Eubacterium eligens* in elderly people varied greatly across different studies. Our results indicated that *Eubacterium eligens* was more abundant in the elderly gut microbiome, which is in line with a study of a healthy Thai cohort;^61^ however, this is contrary to the Japanese population study in which a reduction in *Eubacterium eligens* was observed in elderly people.^62^ These inconsistent observations may be attributed to interpopulation variability, lifestyle differences, and differences in experimental designs and analysis methodologies.

Moreover, we found that *Eubacterium eligens* was associated with multiple features from different omics data. For example, *Eubacterium eligens* had a strong positive correlation with triglycerides (correlation = 0.819, Figure 3b), thus linking our findings with a previous report that *Eubacterium eligens* greatly contributed to CDP diacylglycerol biosynthesis^62^ and implying that *Eubacterium eligens* is closely associated with the metabolism of lipids, which is a class of molecules known to change with aging.^63^ This intriguing finding demonstrated that our analysis uncovered potential gut metagenome-metabolome links influenced by aging.

We identified multiple oral microbiome-derived age-related signatures, most of which were reported to be associated with polymicrobial diseases such as dental caries and periodontitis.^54,55,64,65^ The oral species *Bifidobacterium dentium* was found to be more abundant in elderly people,^66,67^ and it showed high predictive value in the XGBoost model to distinguish between the elderly and young groups. *B. dentium* is an opportunistic cariogenic pathogen which can metabolize carbohydrates and produce acids under physiological conditions in the oral cavity.^68^

Daily oral hygiene habits (e.g., frequency of toothbrushing, efficacy of plaque removal) are constantly influencing the oral microflora throughout life. Toothbrushing and flossing can be powerful means to disrupt plaque and maintain healthy oral ecosystems. It was found that individuals who brush their teeth once per day have a higher Shannon index than those who brush more than once per day.^69^ Poor oral hygiene might lead to an overgrowth of pathogenic microorganisms and the entry of oral microorganisms to the lower respiratory tract.^70^ Multiple studies indicating oral health and oral hygiene behavior have a direct impact on the oral microbiome^71,72^ and influence the balance of gut microbiome.^73^ Oral hygiene habits should be taken into account for future microbiome studies, especially oral microbiome studies.

Another important finding was the age-dependent interactions between oral species and other multiomics features, including gut species, metabolites and phenotypic parameters, demonstrating that microbes participate in regulating host health via complex crosstalk with metabolic activity and environmental factors. For example, the negative correlation between oral *Prevotella melaninogenica* and gut *Eubacterium eligens* may enable us to inspect possible communication routes, including potential competitive relationships or negative feedback, between the oral microenvironment and the distant digestive system. Additionally, two age-related oral species (*Rothia dentocariosa* and *Streptococcus milleri*) were negatively correlated with the MET score, which suggested that lifestyle could affect age-associated oral-derived microbes. Thus, it is apparent that lifestyle changes, especially exercise interventions, may influence the aging process, thereby necessitating further investigations of novel effective strategies for healthy aging.

We further estimated the impact of multiple potential factors including BMI and dietary protein, fat and carbohydrates intakes on the microbiome of participants in our dataset. First, to evaluate the influence of BMI on the microbiome structure, we categorized the individuals according to the definition of the Centers for Disease Control and Prevention classification (https://www.cdc.gov/healthyweight/assessing/bmi/adult_bmi/index.html): normal (18.5 kg/m2 ≤ BMI ≤ 25 kg/m2), overweight (BMI >25 kg/m2), underweight (BMI <18.5 kg/m2). The result illustrates that there was no significant association between BMI and age groups (χ^2^ test, *p* = .294, Table S12) based on BMI stratification. In addition,, we investigated the explained proportion of variance of the microbiome community structure by BMI using PERMANOVA. The variance explained was 0.02 (for both gut and oral microbiomes) and the BMI did not statistically influence the microbiome structure (both *p* > .05 for both gut and oral microbiomes). Therefore, we conclude that BMI was compared between the two age groups and had negligible influences on the community structure in our study.

Subsequently we compared the quantity of daily caloric intake, protein, fat and carbohydrate intakes between elderly and young groups, stratified by daily physical activity level (participants with MET score > 500 min/week were classified as “active”, otherwise labeled as “inactive”). As a result, no statistically significant difference in the daily caloric intake, protein, fat or carbohydrate intakes between the young and elderly groups was observed for all the comparisons (Supplementary Figure S12, all Bonferroni-corrected p-values >0.5, Wilcoxon rank-sum tests), indicating the overall balanced dietary covariates between the young and elderly groups in our study. Furthermore, we assessed the influence of diet on microbiome composition using permutational multivariate analysis of variance (PERMANOVA). None of the dietary factors was significantly associated with the variability of the gut or oral microbiome composition (*p* > .05, 1000 permutations), and these factors cumulatively explained only 3.62% and 5.15% of the variability of the gut and oral microbiome, respectively. Taken together, the dietary factors were overall balanced between two age groups and imposed a negligible influence on the microbiome structure in our study. Exploring age-related alteration of the microbiome in a much larger cohort would allow us to investigate aging-associated microbial signatures specific to different dietary habits.

Additionally, we related our findings with previously identified metabolites in serum and urine metabolome profiles from the same subjects.^74^ Herein, we examined the common key metabolites with significant differences in concentration between the two age groups in the serum (or urine) and intestinal tract. One major finding is the consistent decrease in branched-chain amino acids (BCAAs: valine, leucine, isoleucine) in the gut and serum metabolome of elderly individuals, in accordance with some previous studies showing that BCAAs have a beneficial effect on aging.^30,31^ Dietary BCAAs can be absorbed from the intestines, and their catabolism occurs initially in skeletal muscles and is degraded further in the liver, kidneys, heart, muscle, adipose tissue and brain.^75^ In addition, other important differential metabolites, including N-acetylglucosamine (GlcNAc) and acetic acid (a SCFA that circulates systemically in the host in significant amounts), were shared by the gut and urine metabolome. These findings support that the gut microbiome and its metabolites potentially affect host metabolism. In addition, our statistical analyses identified microbe-metabolite associations, including a negative correlation between *Desulfobulbus oralis* and acetic acid and positive correlations including *Prevotella fusca* – N-acetylglucosamine and *Rothia dentocariosa* – triglycerides, which have been insufficiently described in previous studies.In summary, our study demonstrated that multiomics and integrative analyses based on the gut metabolome, serum metabolomes, urine metabolomes, oral microbiome and gut microbiome would deepen our mechanistic understanding of the microbe-mediated aging process. The common altered metabolites in these metabolomes further reveal that the gut microbiome potentially interacts with host metabolism.

The biological implication and interpretation of the gut microbiome composition for human health is still hindered by certain technical aspects. For example, the taxonomic and functional repertoire of the intestinal microbial system have been insufficiently characterized due to the incompleteness of microbial reference genomes. Encouragingly, with the rapid increase in cultured and uncultured genomes, especially metagenome-assembled genomes (MAGs), the reference microbial genome repertoire of the human gut/oral microbiota has been massively expanded and improved the accuracy of the taxonomic assignments of microbial genomes. For example, several *Eubacterium rectale* isolates were recently reclassified as *Agathbacter rectalis* based on phenotypic, chemotaxonomic and multiple-marker phylogenetic results.^76^ Moreover, Parks et al.^77^ developed the genome taxonomy database (GTDB), which aims to establish a standardized microbial taxonomy based on genome phylogeny. In this database, an average nucleotide identity (ANI)-based approach was adopted to reclassify species, which resulted in the reclassification of several *E. coli* strains to *Shigella* species. These attempts, especially GTDB-based methods, offer a promising direction to obtain high-resolution and high-accuracy taxonomic identification for microbial species. In addition, the microdiversity within the same species should be considered in future studies. We need to deliberately characterize the health-associated strains of the key age-related species, and uncover the functionally relevant strain-level variation related to the aging or other phenotypes.

This study has several limitations. One intrinsic limitation of this study is the sampling in males only. Gender is an important factor potentially influencing the gut microbiome.^78,79^ The reason why we recruited male adults only in this study is that vast number of covariates and confounding factors, including BMI, dietary habits, and clinical features, make the covariates balancing/matching between men and women very challenging and even unrealistic regarding our sample size. Without sufficient covariates matching, the statistical patterns of microbiome change or the microbial associations with aging will be uncomparable between males and females, or between two age groups. Considering the scope, comprehensiveness of data (multimicrobiome, metabolome and clinical features) and sample size we have, we believe that controlling the sex factor by recruiting men only will be a realistic solution for robust pattern recognition in this study. Future in-depth studies require a much larger sample size, including both sexes, a variety of BMI groups, and diversified dietary groups. Notably, some of the alterations in the human microbiome and metabolome we identified might be attributed to interindividual phenotypic variations in different age stages in a Singapore population. In this study all the participants are Chinese Singaporeans. Thus future studies incorporating other ethnic minorities would make the results more unbiased. Except the limitation in sampling, age-associated changes were explored based on “young” and “elderly” groups, which may not reflect continuous changes in the microbiome, metabolic signature, and other phenotypes with aging. Aging is essentially a gradual and progressive process that results in degenerative physiological function. We believe that increasing the sample to include people across a wide age range and adding more age groups would be supportive of capturing the sequential changes with aging. Besides, the microdiversity within the same species should be considered in the future study. We need to well characterize the health-associated strains of the key age-related species and uncover the functionally relevant strain-level variation with the human microbiome. Additionally, to further illustrate the underlying mechanisms of human-microbe interactions, our discovered multiomics signatures and their associations should be experimentally validated with a series of well-controlled *in vitro* assays and animal models.

## Conclusions

Through an integrative multiomics analysis, we identified age-associated changes in the gut and oral microbiomes, as well as important metabolites mediating host-microbe interactions in Singaporeans. Elderly subjects had a higher level of alpha diversity in the gut microbiome than young people. Moreover, compared to the young group, the elderly group harbored 8 gut and 13 oral species that were differentially abundant, including elevated levels of gut *Eubacterium eligens* and oral *Bifidobacterium dentium* and depletion of gut *Flavonifractor plautii*, oral *Prevotella melaninogenica* and oral *Prevotella fusca*. Furthermore, we identified several potential profound interactions among microbes, metabolites and host phenotypic characteristics, such as a positive correlation between *Eubacterium eligens* and triglycerides and a negative correlation between oral species (*Rothia dentocariosa* and *Streptococcus milleri*) and the metabolic equivalents (MET) score. These findings demonstrated the complex host-microbe-metabolite interplay. Nevertheless, future studies are required to shed further light on the aging process.

## Materials and Methods

### Participants and sample collection

The study participants and methods of sample collection have been reported previously.^80^ Briefly, 32 healthy young (age, 23.1 ± 1.4 years) and 30 generally healthy elderly (age, 69.0 ± 3.5 years) Chinese male Singaporeans were recruited. Dietary records (2 weekdays and 1 weekend) were analyzed using a curated in-house database that was compiled from various online sources and nutrition labels (See Supplementary Table S13). Basic health screen including resting heart rate, blood pressure, body mass and height were measured. For blood sample collection, overnight fasting venous blood was drawn from each participant using the BD Vacutainer tubes (BD, Singapore) by registered nurses, and then centrifuged to separate plasma and serum and aliquoted according to manufacturer’s instruction. Part of whole blood and serum samples were sent for whole blood hematology determinations, lipid profile, wr-CRP and glucose fasting analysis in Quest Laboratories Pte Ltd (Singapore). Cytokines including IL-6, IL-7, IL-8, MCP-1(MCAF), MIP-1b, TNF-a etc. were analyzed using Bio-Plex Pro™ Human Cytokine 17-plex Assay kit (Bio-Rad, #M5000031YV) in Bio-Plex 200 system (Bio-Rad, Singapore). The rest was stored at −80°C freezer until further analysis. Mid-stream urine was self-collected by the participant according to provided instructions. The collected urine samples were stored at −80°C with adding 0.1% sodium azide.

For feces collection, participants were instructed to defecate directly into 650 mL sterile snapped on lid containers (Fisherbrand™ Commode Specimen Collection System, Fisher scientific, Singapore) to prevent contaminations by toilet water and materials. Fresh fecal samples were placed in a ziplock bag with an AnaeroGen^TM^ pack (2.5 L, Thermo scientific, Singapore) for transportation to the laboratory under anaerobic conditions for a maximum of 3 h. Samples were aliquoted into screw-cap tubes under anaerobic conditions, and then stored at −80°C until processed.

For saliva collection, participants first rinsed their mouth with water to remove any food residues and then rested for at least 10 minutes before providing a saliva sample. Passive oral drool was used for saliva collection. Saliva samples collected in cryogenic micro tubes were stored at −80°C until processed.

### DNA extraction, library preparation, and metagenomic sequencing

For each fecal sample, 0.25 g aliquot was extracted using Qiagen Fast Stool DNA isolation kit with slight modification. In the first lysis step with InhibitEx Buffer, 0.4 g of silica beads (0.1 mm 0.2 g and 1.0 mm 0.2 g) was added into the sample prior to homogenization in FastPrep®-24 (MP Biomedicals) for 1 minute at 4 m per sec. Library preparation was performed according to Illumina’s TruSeq Nano DNA Sample Preparation protocol. The samples were sheared on a Covaris E220 to ~450bp, following the manufacturer’s recommendation, and uniquely tagged with one of Illumina’s TruSeq HT DNA barcodes to enable sample pooling for sequencing. Finished libraries were quantitated using Promega’s QuantiFluor dsDNA assay and the average library size was determined on an Agilent Tapestation 4200. Library concentrations were then normalized to 4 nM and validated by qPCR on a QuantStudio-3 real-time PCR system (Applied Biosystems), using the Kapa library quantification kit for Illumina platforms (Kapa Biosystems). Libraries were then pooled at equimolar concentrations and sequenced on an Illumina HiSeq2500 sequencer in rapid mode and a read-length of 251bp paired-end (Illumina V2 Rapid sequencing reagents).

For each saliva sample, a volume of 1 mL was extracted using Qiagen Blood Mini Kit according to the manufacturer’s instructions. Libraries from saliva DNA were prepared with the Accel-NGS 2S Plus DNA Kit (Swift Biosciences), following the manufacturer’s instructions. Libraries were either prepared manually or automated on a Bravo NGS Workstation (Agilent). The starting amount of DNA for library preparation was normalized to 5ng. DNA shearing was performed on either a Covaris S220 or E220 focused-ultrasonicator with the following settings: Peak Power: 175, Duty Factor: 5.0, Cycles/Burst: 200, Run Time: 90s. All libraries were dual-barcoded, using the 2S Dual Indexing Kit (Swift Biosciences). For PCR amplification, which selectively enriches for library fragments that have adapters ligated on both ends, the PCR cycles were normalized to 8 for all libraries with a starting amount of 5ng of DNA.

Finished libraries were quantitated using Promega’s QuantiFluor dsDNA assay and the average library size was determined on an Agilent Tapestation 4200. Library concentrations were then normalized to 4 nM and validated by qPCR on a QuantStudio-3 real-time PCR system (Applied Biosystems), using the Kapa library quantification kit for Illumina platforms (Kapa Biosystems). Libraries were then pooled at equimolar concentrations and sequenced on an Illumina HiSeq2500 sequencer in rapid mode and a read-length of 251bp paired-end (Illumina V2 Rapid sequencing reagents).

### Metagenomic data pre-processing

The quality control of raw reads was performed by removing adaptor regions, low quality bases/reads and PCR-duplicated reads as previously described,^81,82^ the script is available on GitHub (https://github.com/TingtZHENG/VirMiner/). Afterward, the high-quality reads were mapped against human reference genome using Burrows-Wheeler Aligner (BWA)^83^ to filter out the host contamination. The mapped reads which met two criteria were removed: 1) consecutive exact match ≥ 25 bp; 2) identity ≥ 95%.

### Microbial taxonomic profiling

Taxonomic profiling of gut microbiome was performed using MetaPhlAn3.0 (https://github.com/biobakery/MetaPhlAn/tree/3.0),^84^ to classify reads into different taxonomies and determine taxonomic relative abundances profiles, with default parameters. A total of 268,004,732 high-quality paired sequences were obtained from stool samples, with 4,322,657 ± 950,303 (average ± standard deviation) reads per sample, which were assigned into 256 OTUs and classified into 99 taxa at the genus level. Taxonomy assignment of oral microbiome was done by Kraken 2^48^ (https://ccb.jhu.edu/software/kraken2/), with the Minikraken database (version minikraken_8GB_20200312) downloaded from ftp://ftp.ccb.jhu.edu/pub/data/kraken2_dbs/. The taxonomic relative abundance was calculated using Bracken (version 2.5). The OTU profiling was firstly randomly rarefied to include the same number of sequences per sample and then the relative proportion of each taxa was calculated. Finally, we generated a data set containing 59,496,988 high-quality paired sequences with an average sample read counts of 959,629 ± 872,602, which were classified into 5,217 OTUs and 1,473 taxa at the genus.

### Microbial diversity analysis

The alpha diversity was calculated using Shannon index.^85^ The species richness was measured using Chao1 estimator. Wilcoxon rank sum tests were used to evaluate the differences in alpha diversity and species richness between elderly and young groups. For beta diversity analysis, the nonmetric multidimensional scaling (NMDS) plot was based on Bray-Curtis dissimilarities matrix.^86^ ADONIS test was used to determine the overall community composition difference between elderly and young samples. These calculations and visualizations were implemented using the R package vegan.^87^

### Differential abundance analysis

Species present in at least 30% of all the samples were used for differential abundance analysis. IndVal tests^88^ were performed to detect the differentially abundant species between elderly and young groups, followed by adjustment for multiple testing using the Benjamini and Hochberg method. Species with a false discovery rate (FDR) ≤ 0.1 were deemed as significant.

### Species-based classifier

XGBoost algorithm from R package xgboost was performed to discriminate elderly and young groups using the abundance profiles of gut and oral species. The species present in ≥30% of samples were selected to build the XGBoost models based on only gut or oral species. After tuning various parameters, the best performing models were selected. For gut species, the best performing model was derived with the following parameters: nrounds = 100, eta = 0.1, colsample_bylevel = 0.3333333, max_depth = 6, min_child_weight = 1, subsample = 1. For oral species, the best performing model was derived with the following parameters: nrounds = 100, eta = 0.3, colsample_bylevel = 1, max_depth = 4, min_child_weight = 1, subsample = 1. Then combining the important features extracted from the two best performing models to build the final XGboost model. The best performing model based on combined gut and oral species was derived with the following parameters: nrounds = 100, eta = 0.2, colsample_bylevel = 0.3333333, max_depth = 4, min_child_weight = 1, subsample = 0.75. The important features were identified using xgb.importance and their importance scores were normalized to 1. The performance of the XGboost models were quantified by the AUC of the receiver operating characteristic (ROC) with 5-fold cross validation. The generation of ROC and AUC calculation was implemented in the pROC R package.^89^ The highest accuracies the models achieved were also calculated.

### Assembly-free functional annotation of metagenomic data

Ten thousand high-quality reads were randomly subsampled from each sample and mapped to KEGG Orthology (KO) database^90^ using blastx with an *e*-value cutoff of 1e-5. Then these identified KO categories were annotated according to KEGG pathways. Wilcoxon rank sum test was used to identify differentially abundant pathways.

### The association analysis among multiomics datasets

The sPLS-DA implemented in the MixOmics R package^36^ was employed to identify age-dependent connections between gut and oral species from species present in at least 30% of all the samples. Subsequently, the application of sPLS-DA was extended to the multiomics integrative analysis: 1) association study of the age-associated functional capability among gut microbiome, oral microbiome and gut metabolome; 2) the integrative analysis among gut and oral microbiome, metabolites, and important phenotypic parameters. To reduce the dimensionality of the input data to a manageable size, only differential features between young and elderly subjects from multiomics datasets were used, resulting in the identification of a highly correlated multiomics signature panel.

Two parameters were tuned using the function *tune.block.splsda*, namely the number of dimensions and the number of variables to select on each dimension. To identify the links between gut and oral species, the number of selected variables to be retained on each component was chosen over the grid of values, ranging from 4 to 20 with an interval of 1, for both gut and oral species. In terms of multi-omics integrative analysis, the grids of the number of selected variables to be kept for each component were set as ranging from 4 to 8 with an interval of 1 for the gut species, ranging from 4 to 13 with an interval of 1 for the oral species, ranging from 1 to 10 with an interval of 1 for the gut metabolites, ranging from 1 to 5 with an interval of 1 for the phenotypic parameters and ranging from 1 to 2 with an interval of 1 for the lipid profiles. The correlations between variables were identified using the function *circos*. The P-value of each correlation was calculated by sample permutations (randomly assigned samples into elderly and young groups) for 100 times, the corresponding FDR adjusted P-value was also calculated.

### Compositional balance analysis

The *selbal* algorithm proposed by Rivera-Pinto et al.^27^ was used to identify microbial signatures that best discriminate the two age groups, with default parameters. The abundance matrix of genera present in at least 30% samples was used for this analysis. The group information was set as the outcome.

### Fecal non-targeted metabolomics analysis

Fecal samples were prepared for metabolomics analysis as previous described GC-MS method^91,92^ with modification. Methanol was added to 250 mg of fecal sample to a final concentration of 200 mg/ml, ribitol (10 ug/ml) was added as an internal standard. Solutions were completely homogenized and then centrifuged at 2530 × g at 10°C for 15 min. A 50 ul aliquot of each extract was dried in an Eppendorf rotary vacuum concentrator and stored at −80°C until derivatization. Derivatization was conducted as follows: methoximation was achieved by adding 40 ul of methoxyamine–HCl (20 mg/ml in pyridine), followed by incubation for 90 min at 30°C with shaking at 1200 rpm. Trimethylsilyl derivatization was carried out by adding 40 ul N-trimethylsilyl-N-methyl trifluoroacetamide (MSTFA) and incubation at 60°C for 45 min. The sample is centrifuged at 12000 rpm for 60 min, and the supernatant was transferred to a glass vial for GC-MS analysis.

GC-MS analyses were carried out as follows: derivatized samples were analyzed by injection (1ul aliquot) with split ration 2:1 into an Agilent Technologies GC-MS system 6890–5973 N. A HP-5 MS capillary column (30 m × 0.250 mm i.d.; film thickness: 0.25 μm; Agilent) was used for separation. Helium was used as a carrier gas at 1.1 mL/min. The initial oven temperature was set to 70°C, then increased to 75°C at 1°C/min, and subsequently increased to 300°C at 5.63°C/min. The data were acquired in a full scan mode from 45–600 m/z.

The PARADISe spectral deconvolution and identification software (Version 1.1.8)^93^ was used to process the total ion chromatogram data. Deconvolution of mass spectra of peaks and integration of areas of deconvoluted peaks were based on PARAFAC2 modeling. Mass spectra identification was performed based on deconvoluted mass spectra using integrated NIST search engine (version 2.0 f) which was linked to the NIST 08 mass spectral library. The compound list was filtered based on match probability over 90%. The relative concentrations of compounds were quantified by comparing the integrals of specific metabolite peak with the integral of the internal standard ribitol.

The Pathway Activity Profiling (PAPi) algorithm^32^ was applied to calculate the activity score of each metabolic pathway annotated in the KEGG database based on the number of metabolites associated with each pathway and their abundances. The significantly differential metabolites and pathways between the elderly and young samples were identified using Wilcoxon rank sum test, followed by FDR correction. The cutoff of FDR adjusted P-value was 0.05.

## Supplementary Material

Supplemental Material

## Data Availability

The raw metagenomic sequences are deposited at NCBI’s BioProject database under accession PRJNA 762543.
