# Supplementary figures and images for: Integrative multiomics analysis reveals host-microbe-metabolite interplays associated with the aging process in Singaporeans
Source: Gut Microbes. 2022 May 12;14(1):2070392. doi: 10.1080/19490976.2022.2070392 (PMC9116421; doi:10.1080/19490976.2022.2070392)

# A

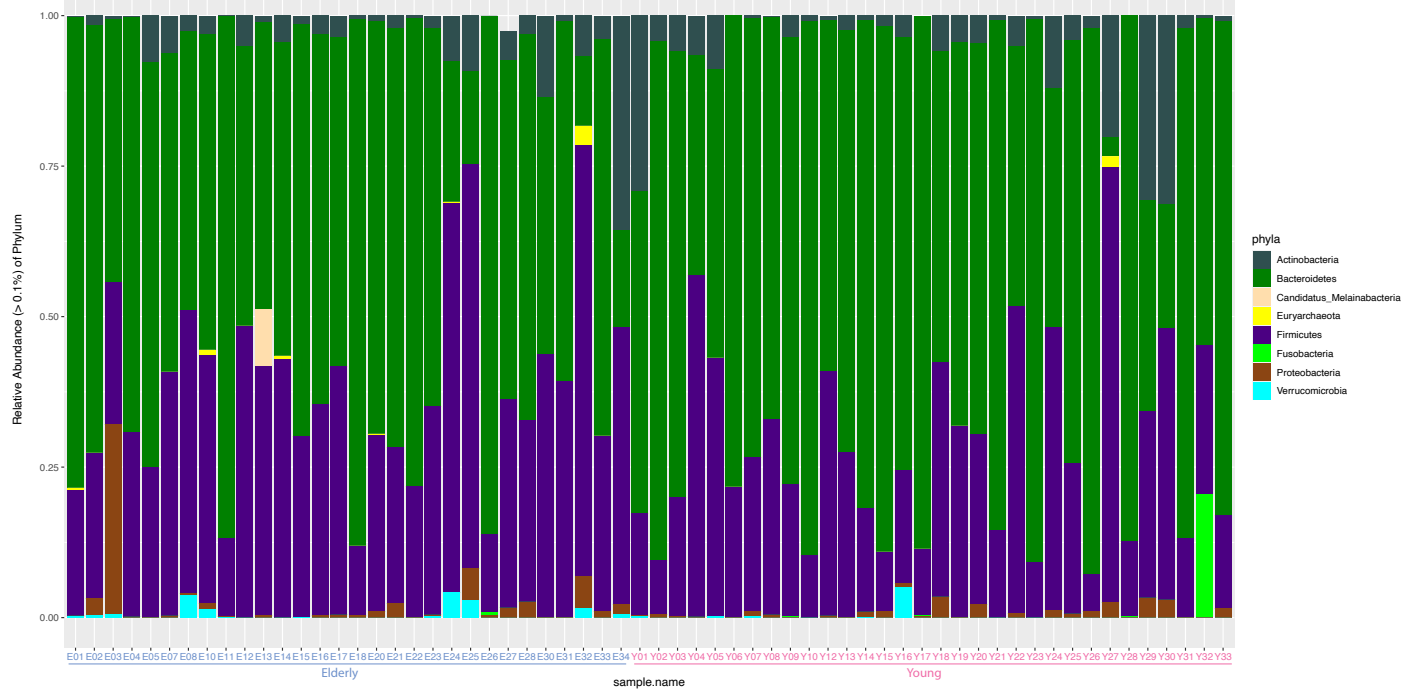

# B

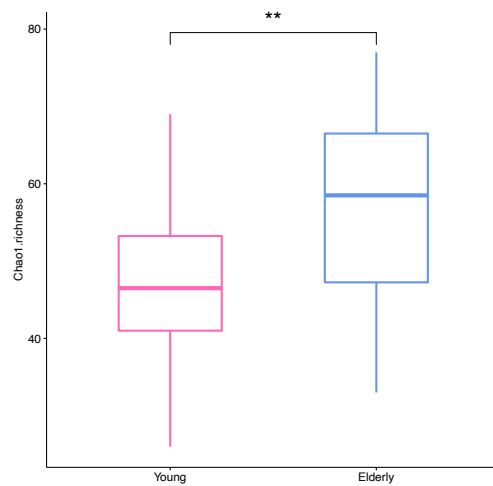

Supplement: Supplemental Material [file KGMI_A_2070392_SM9370.zip › Supplementary Figure S1.pdf]

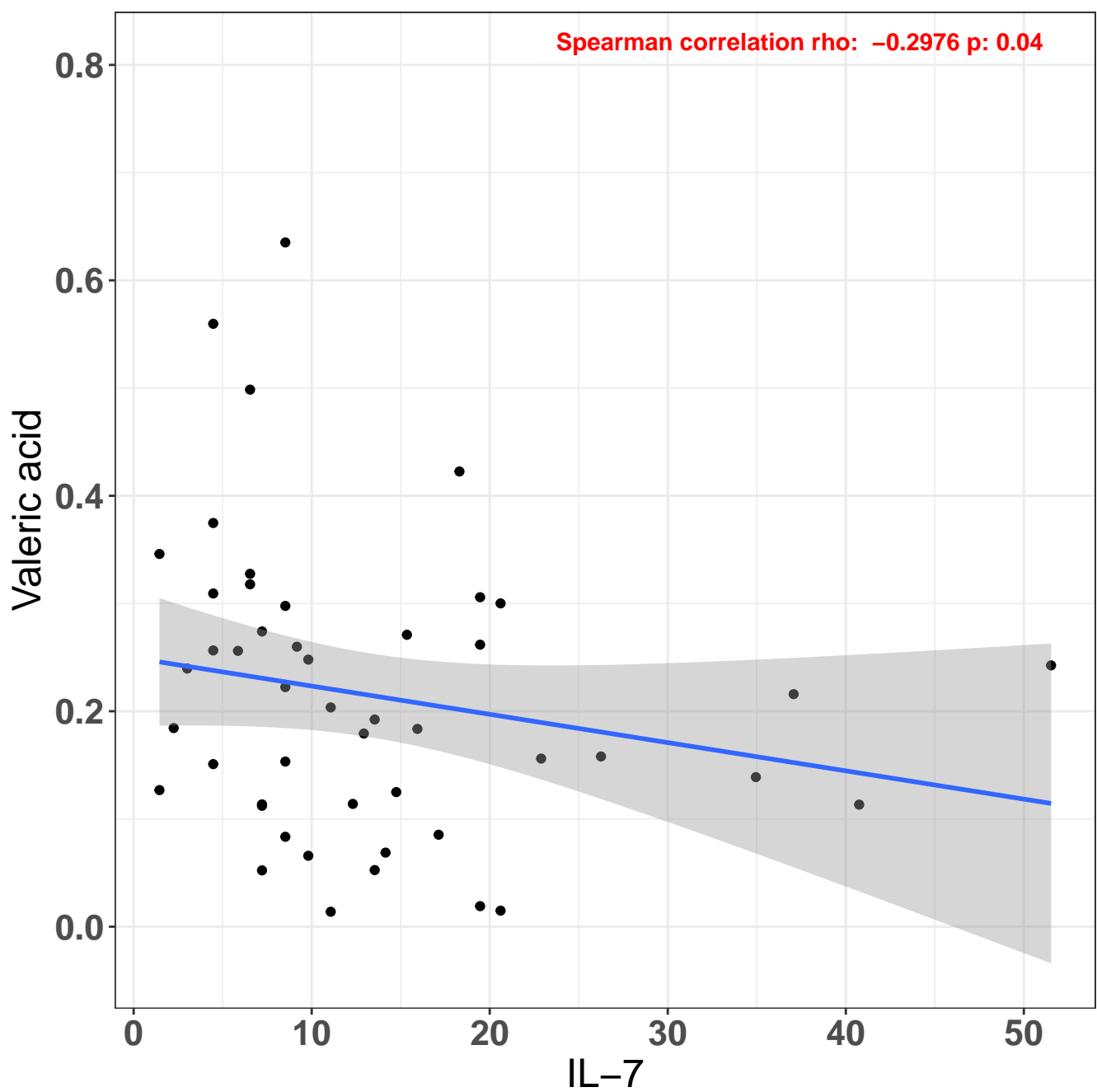

Supplement: Supplemental Material [file KGMI_A_2070392_SM9370.zip › Supplementary Figure S10.pdf]

A

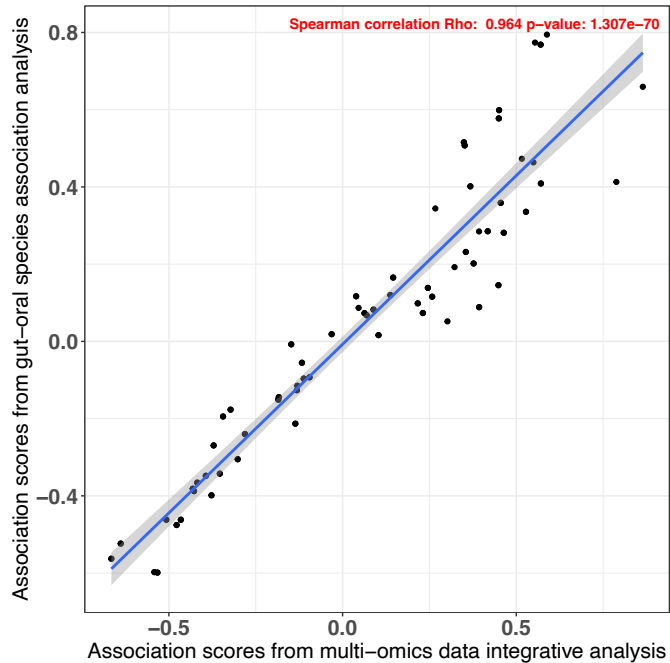

B

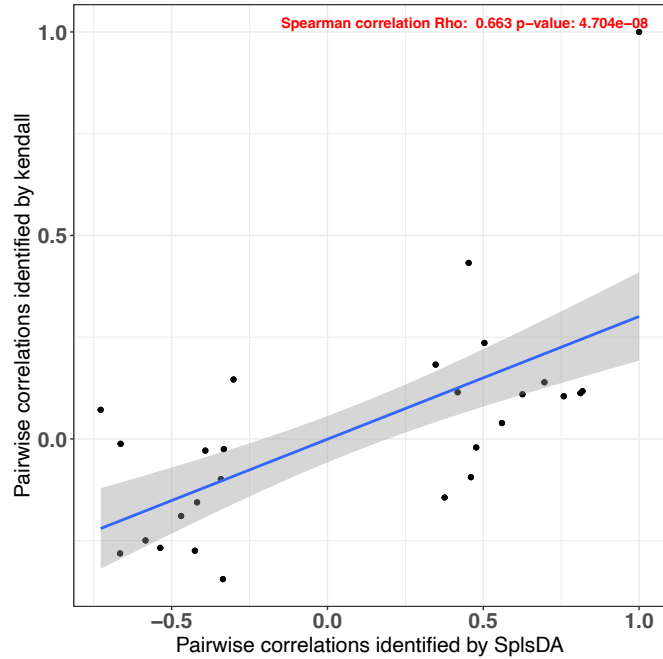

Supplement: Supplemental Material [file KGMI_A_2070392_SM9370.zip › Supplementary Figure S11.pdf]

**A**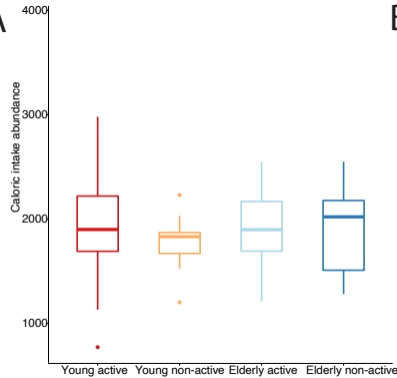**B**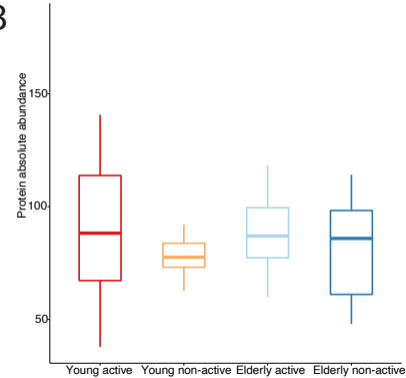**C**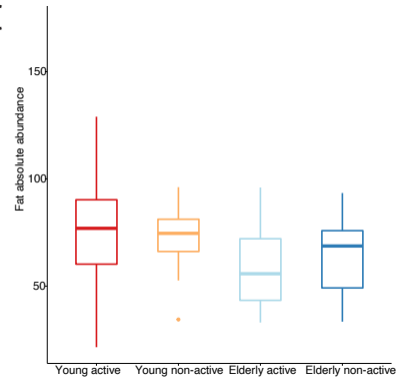**D**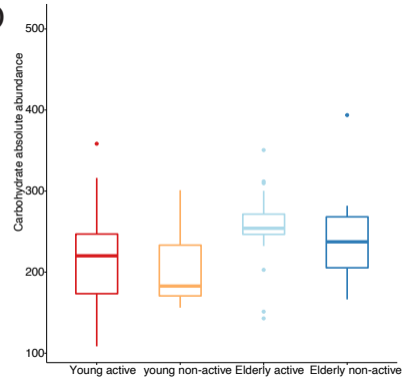

Supplement: Supplemental Material [file KGMI_A_2070392_SM9370.zip › Supplementary Figure S12.pdf]

A

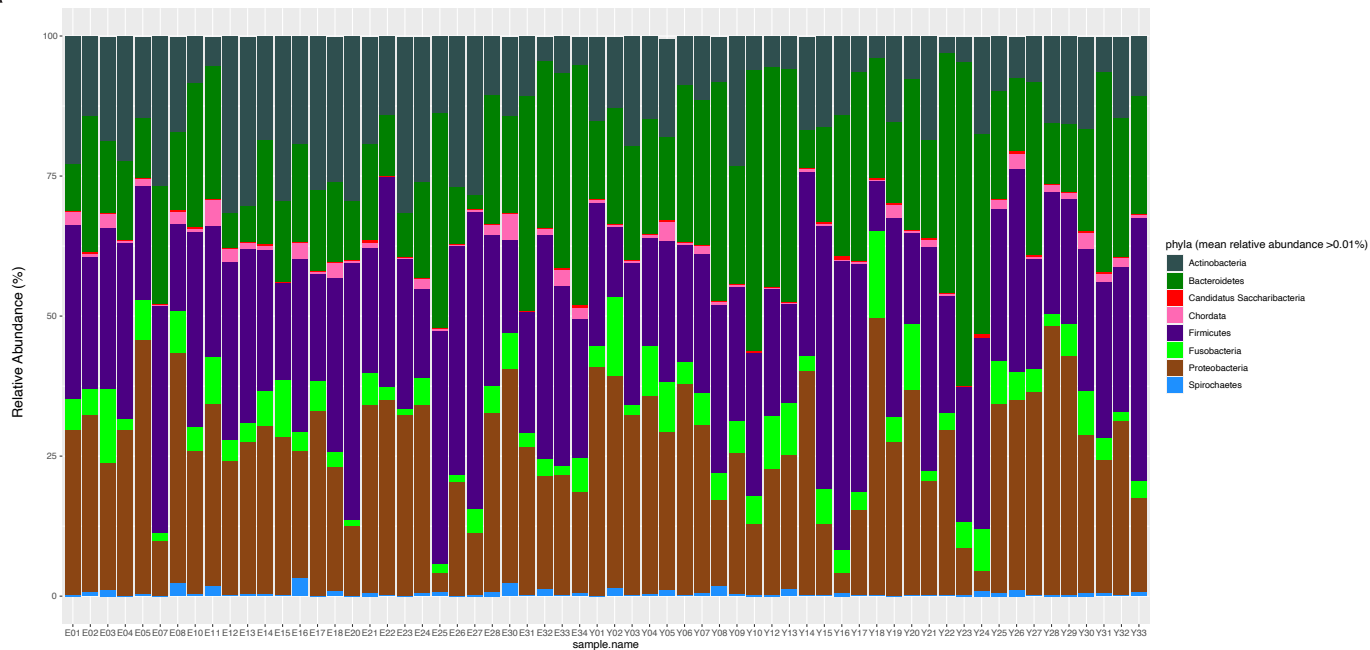

B

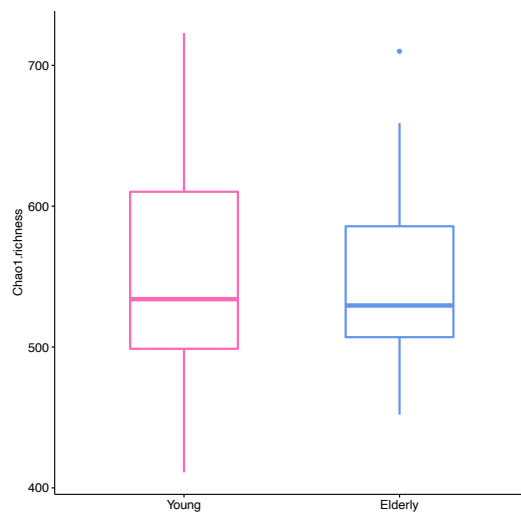

Supplement: Supplemental Material [file KGMI_A_2070392_SM9370.zip › Supplementary Figure S2.pdf]

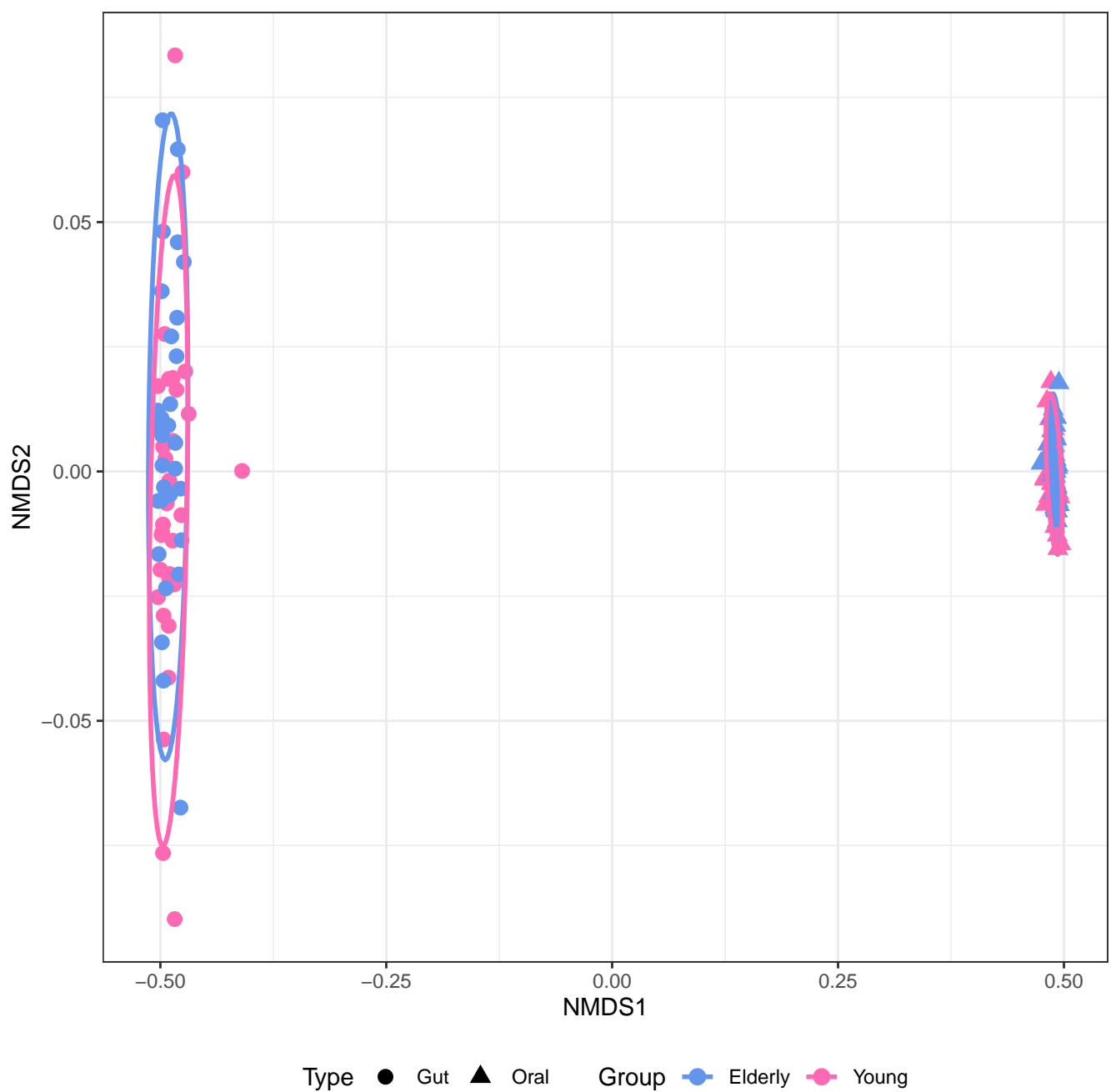

Supplement: Supplemental Material [file KGMI_A_2070392_SM9370.zip › Supplementary Figure S3.pdf]

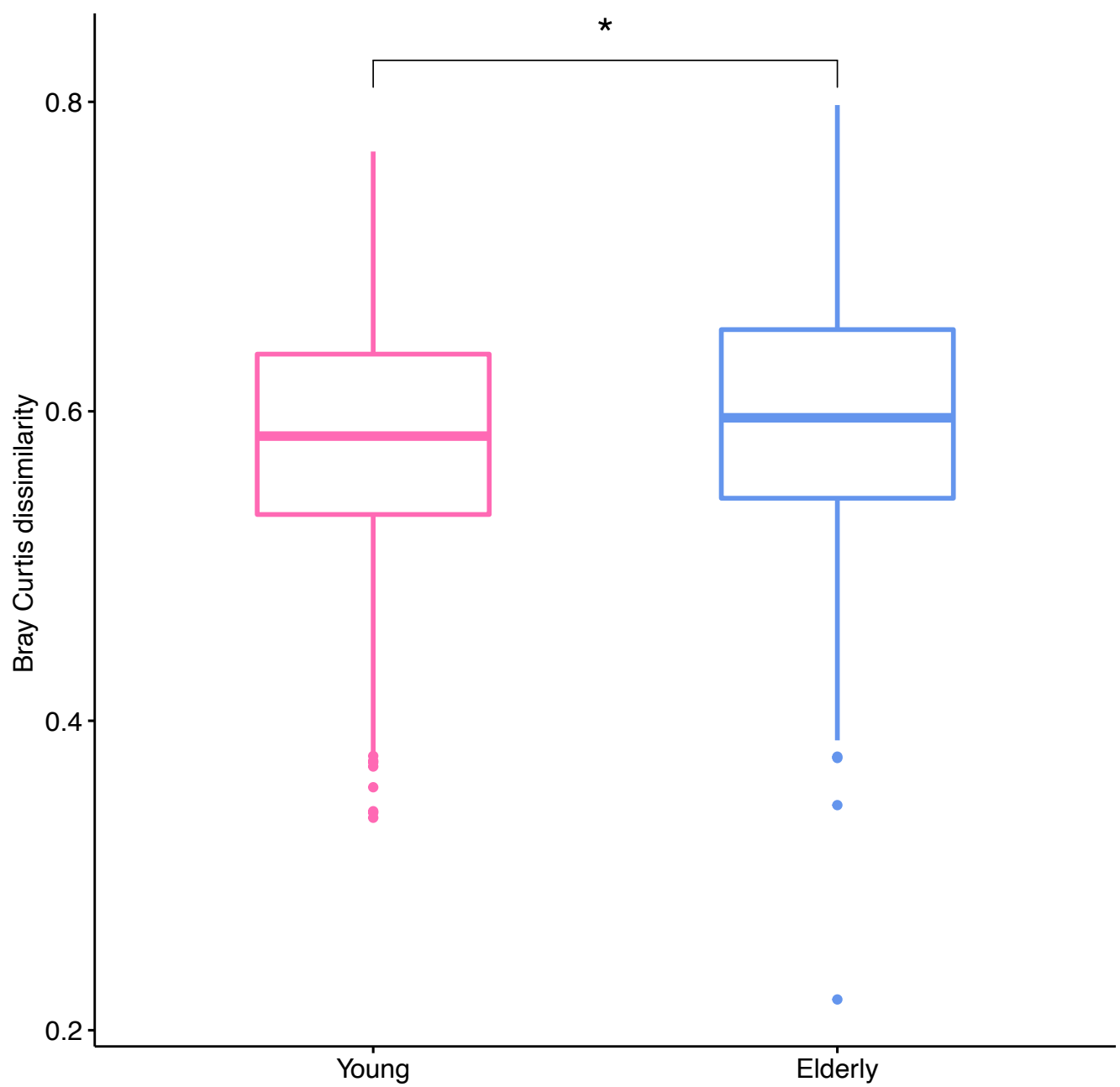

Supplement: Supplemental Material [file KGMI_A_2070392_SM9370.zip › Supplementary Figure S4.pdf]

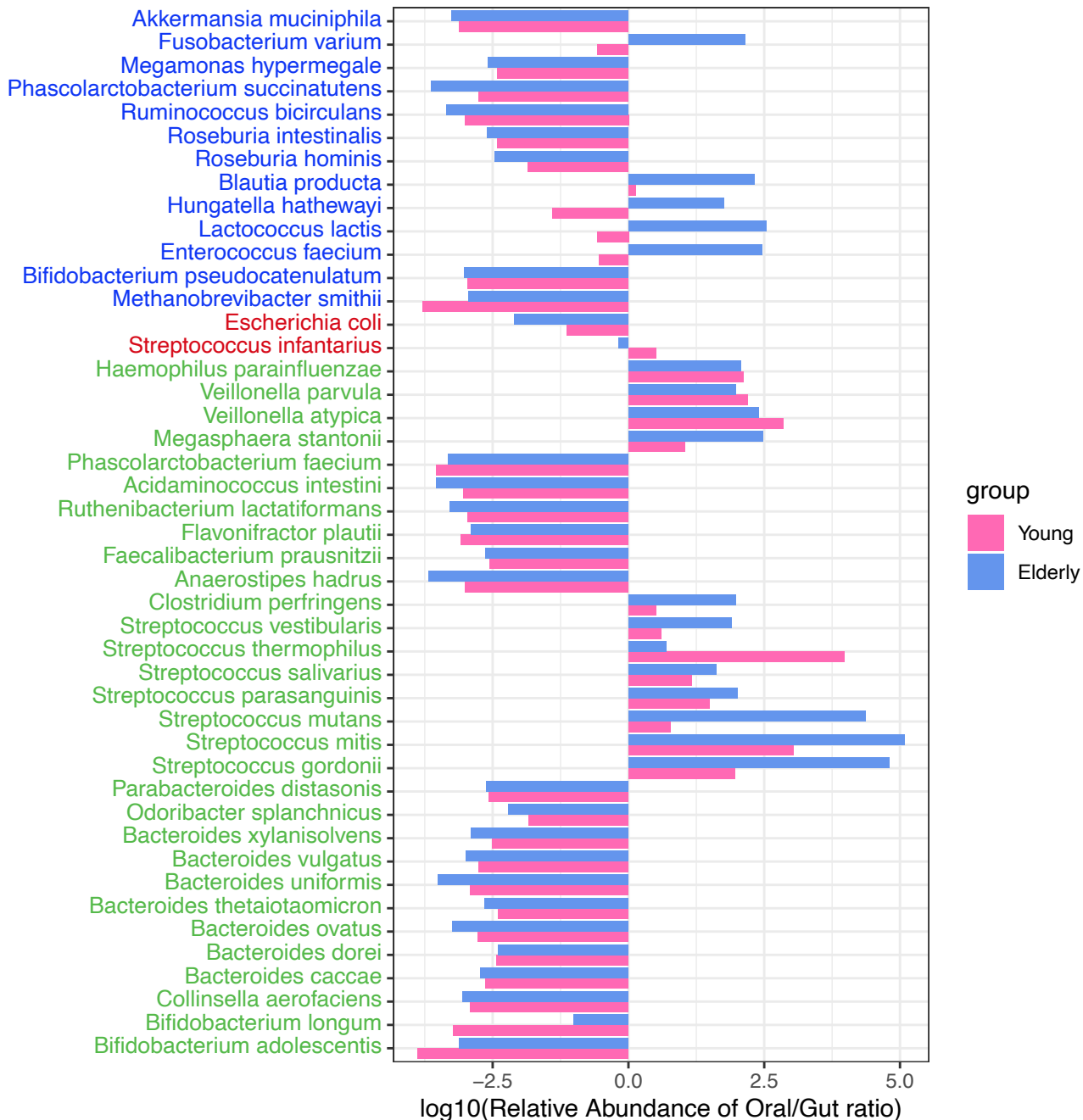

Supplement: Supplemental Material [file KGMI_A_2070392_SM9370.zip › Supplementary Figure S5.pdf]

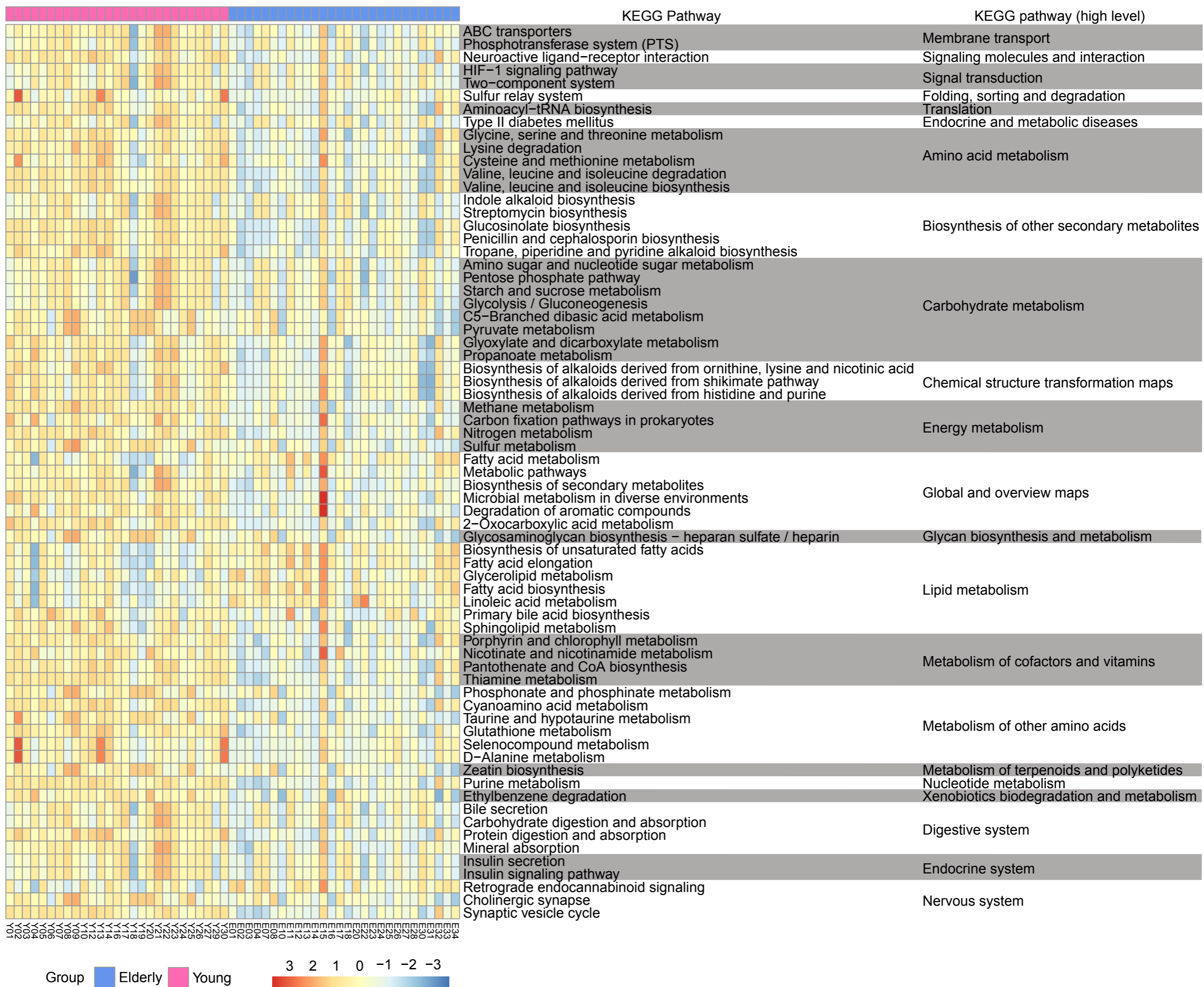

Supplement: Supplemental Material [file KGMI_A_2070392_SM9370.zip › Supplementary Figure S6.pdf]

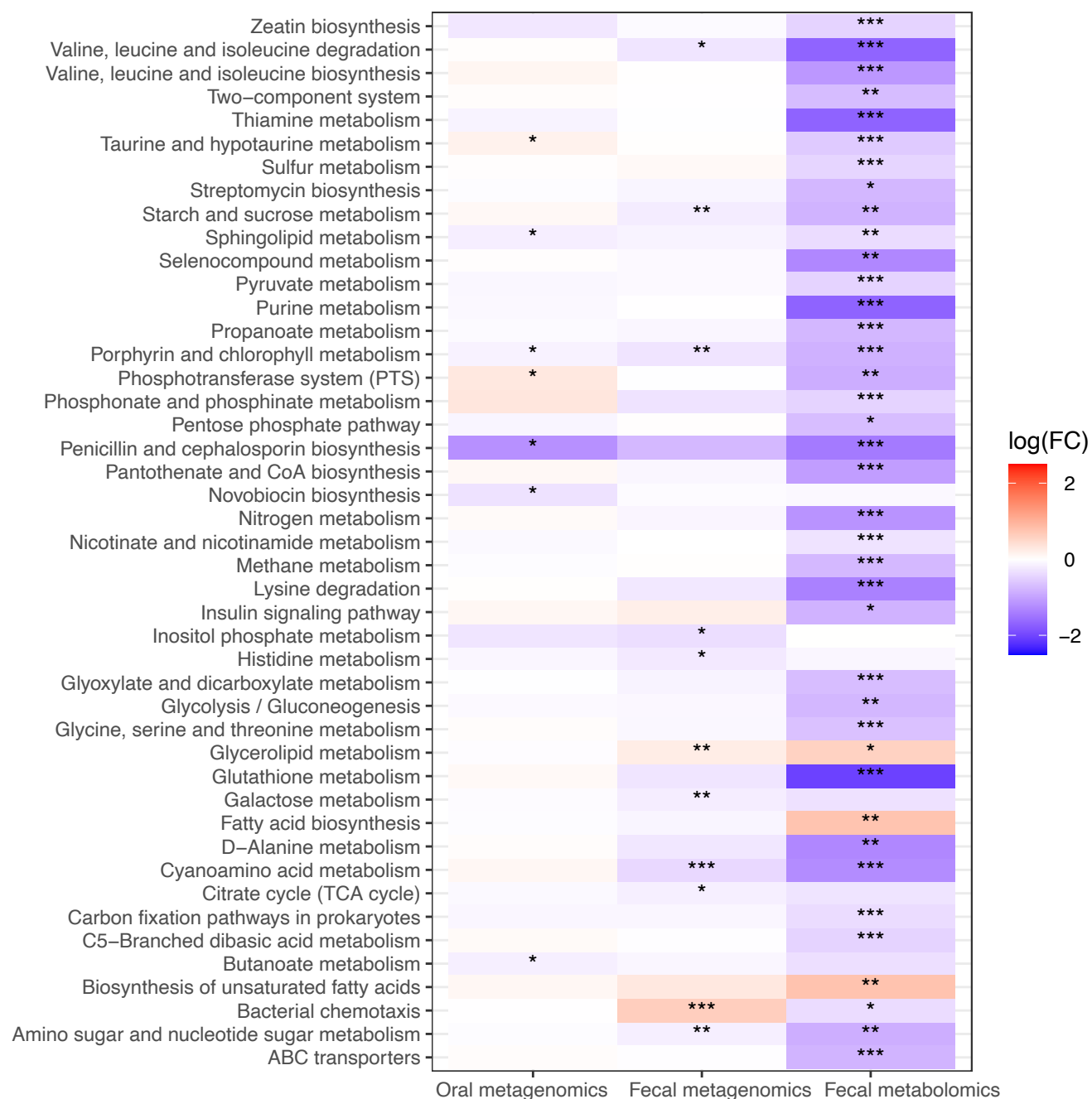

Supplement: Supplemental Material [file KGMI_A_2070392_SM9370.zip › Supplementary Figure S7.pdf]

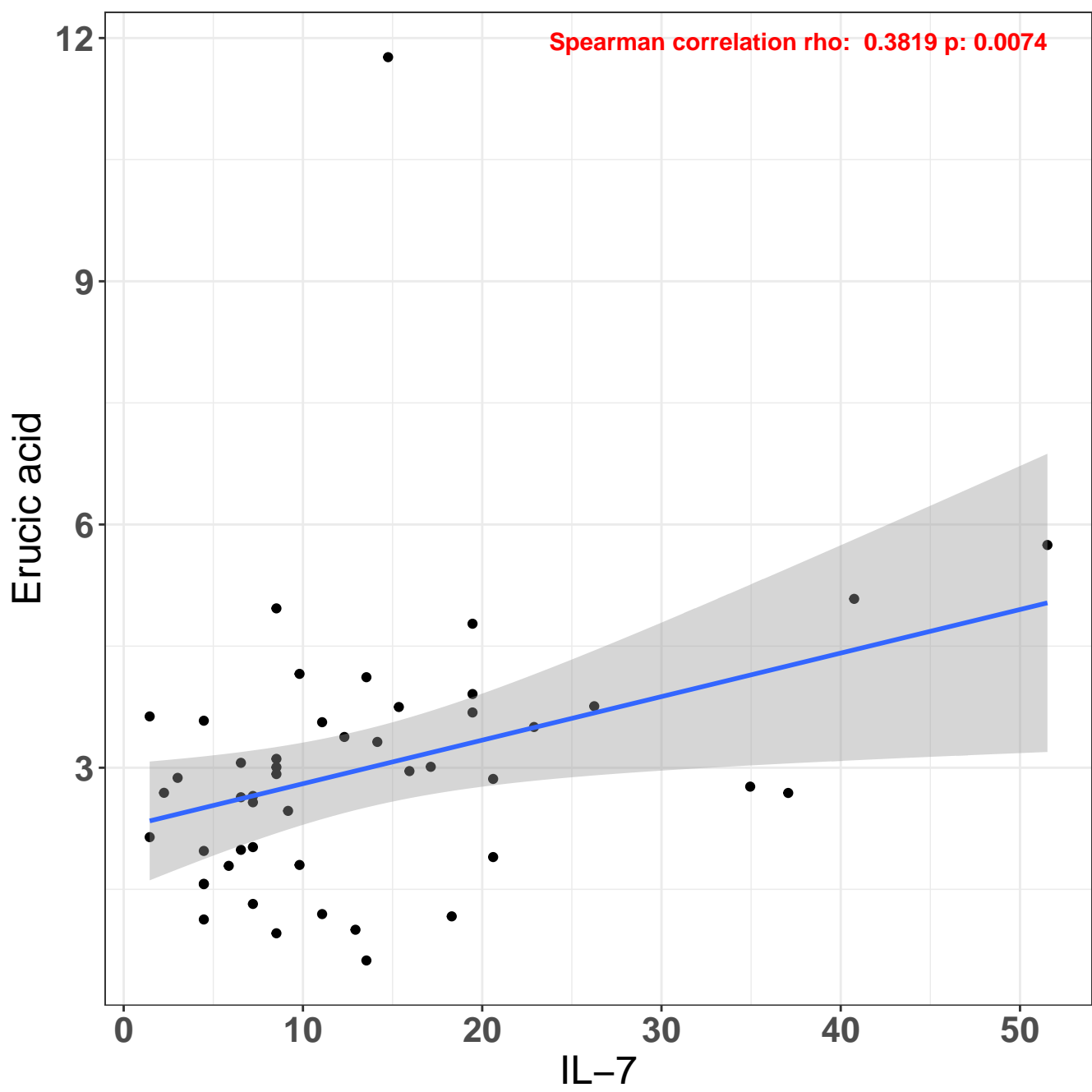

Supplement: Supplemental Material [file KGMI_A_2070392_SM9370.zip › Supplementary Figure S8.pdf]

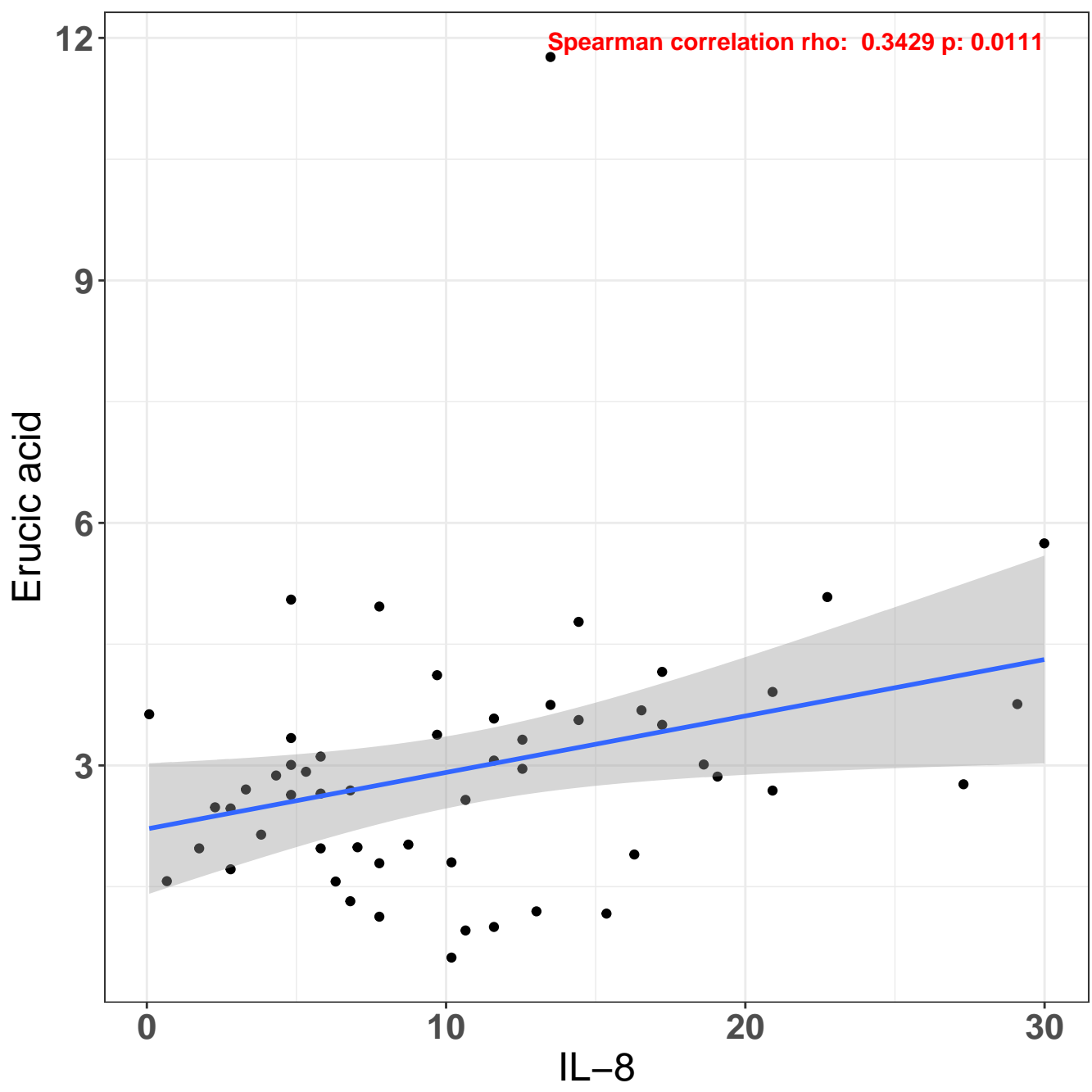

Supplement: Supplemental Material [file KGMI_A_2070392_SM9370.zip › Supplementary Figure S9.pdf]
